# An Injectable Metal‐Drug Supramolecular Condensate Enables Radiosensitization and Systemic Antitumor Immunity for Post‐Surgical Osteosarcoma Therapy

**DOI:** 10.1002/advs.77569

**Published:** 2026-08-31

**Authors:** Tianhua Xiao, Zunlei Gong, Chuke Chen, Hui Yu, Yu Cai, Can Liu, Hongkai Huang, Ying Li, Jinyuan Guan, Le Wang, Chengyun Ning, Guoxin Tan, Lei Zhou

**Affiliations:** ^1^ School of Chemical Engineering and Light Industry Guangdong University of Technology Guangzhou China; ^2^ Department of Orthopaedic Surgery Guangzhou Key Laboratory of Spine Disease Prevention and Treatment The Third Affiliated Hospital Guangzhou Medical University Guangzhou China; ^3^ Department of Spine Surgery Center for Orthopedic Surgery The Third Affiliated Hospital of Southern Medical University Guangzhou China; ^4^ School of Materials Science and Engineering National Engineering Research Center for Tissue Restoration and Reconstruction South China University of Technology Guangzhou China

**Keywords:** immune microenvironment reprogramming, immunogenic cell death, metal‐drug supramolecular condensate, osteosarcoma, radiotherapy sensitization, recurrence and metastasis

## Abstract

Radiotherapy (RT) is widely used as an adjuvant treatment after surgical resection for osteosarcoma, but its efficacy is often limited by insufficient radiosensitization and an immunosuppressive tumor microenvironment that restricts systemic antitumor immunity and promotes recurrence and metastasis. Here we report an injectable metal‐drug supramolecular condensate formed from hafnium ions and alendronate (Hf‐ALN) and loaded with the COX‐2 inhibitor celecoxib (CXB) for synergistic radioimmunotherapy of postoperative osteosarcoma. The condensate forms through coordination‐driven liquid‐liquid phase separation between Hf^4+^ ions and alendronate, enabling local retention within postoperative bone defects and sustained therapeutic release. Upon X‐ray irradiation, the Hf component amplifies radiation energy deposition and reactive oxygen species generation, thereby enhancing radiation‐induced DNA damage and immunogenic cell death. Meanwhile, the acidic tumor microenvironment triggers responsive release of CXB and ALN; CXB inhibits the COX‐2/PGE_2_ immunosuppressive pathway, while ALN promotes macrophage repolarization toward the M1 phenotype, collectively remodeling the tumor immune microenvironment. This strategy enhances dendritic cell maturation, activates cytotoxic CD8^+^ T cells, and reduces regulatory T‐cell infiltration. In orthotopic osteosarcoma models, CXB@Hf‐ALN combined with RT suppresses tumor recurrence, preserves bone integrity, inhibits pulmonary metastasis, and prolongs survival with minimal systemic toxicity, offering a promising strategy to improve postsurgical RT outcomes in osteosarcoma.

## Graphical abstract

This work designed an injectable metal‐drug supramolecular condensate formed from hafnium ions and alendronate (Hf‐ALN) and loaded with the COX‐2 inhibitor celecoxib (CXB) for synergistic radioimmunotherapy of postoperative osteosarcoma. Mechanistically, upon X‐ray irradiation, Hf enhances radiation energy deposition, ROS generation, and DNA damage, while CXB blocks COX‐2/PGE_2_ and ALN repolarizes M1 macrophages, remodeling immune microenvironment to inhibit recurrence/metastasis and preserve bone integrity.

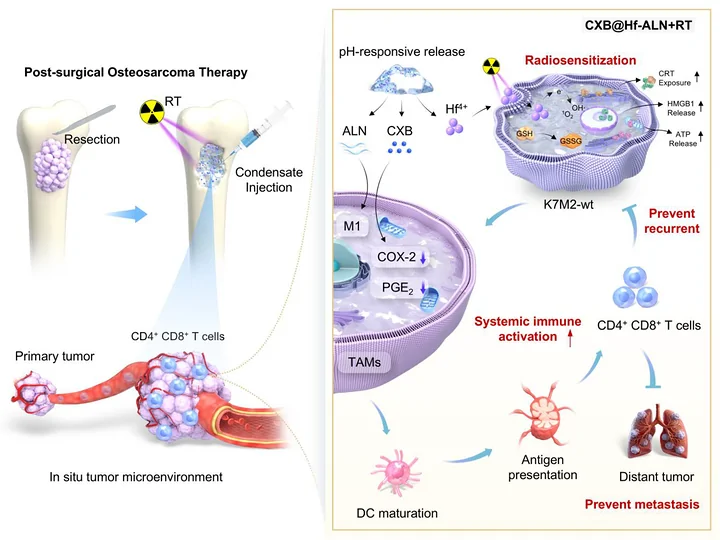

## 1 Introduction

Osteosarcoma (OS) is the most common primary malignant bone tumor and predominantly affects children and adolescents [1, 2, 3]. Despite the introduction of multi‐agent chemotherapy combined with surgical resection, therapeutic progress has remained largely stagnant over the past four decades. Patients with localized disease achieve long‐term survival rates of approximately 60–70%, whereas those with metastatic or recurrent disease have markedly poorer outcomes [4]. Pulmonary metastasis is the most frequent pattern of dissemination and represents the leading cause of osteosarcoma‐related mortality. Even with aggressive multimodal therapy, approximately 30%–40% of patients eventually develop recurrence or metastasis, and this risk increases to nearly 80% in patients presenting with metastatic disease at diagnosis. These clinical realities underscore an urgent need for therapeutic strategies capable of simultaneously eliminating residual local tumor cells and preventing systemic metastatic progression.

Radiotherapy (RT) is commonly used as an adjuvant strategy to prevent recurrence after osteosarcoma resection, however, its therapeutic efficacy remains limited [5, 6, 7]. The antitumor effects of RT are largely restricted to the irradiated region, and conventional radiation doses often fail to penetrate the dense mineralized bone matrix effectively, resulting in insufficient energy deposition within tumor tissues [8]. Although dose escalation can partially improve local tumor control, excessive irradiation may disrupt immune homeostasis and promote tumor progression and metastasis through remodeling of the tumor immune microenvironment [9]. Consequently, combining RT with immunotherapies such as immune checkpoint inhibitors (ICIs) has attracted increasing attention, yet effective synergistic strategies remain elusive [10]. In this context, high atomic number (high‐Z) nanomaterials—including gold, hafnium, and lanthanides—have emerged as promising radiosensitizers that amplify radiation energy deposition and enhance immunogenic tumor cell death [11, 12]. Among them, hafnium (Z = 72) has entered clinical investigation owing to its favorable physicochemical properties [13, 14]. However, most hafnium‐based nanomaterials are rapidly cleared in vivo, limiting tumor retention and often requiring repeated administration [15]. Therefore, developing hafnium‐based radiosensitizing systems capable of in situ long‐term retention and sustained therapeutic activity remains critical for improving RT‐based osteosarcoma therapy.

Tumor‐associated macrophages (TAMs) are the dominant immune cells in the osteosarcoma tumor microenvironment, accounting for more than 50% of infiltrating immune cells [16]. Surgical inflammation further recruits TAMs to the resection site, where they accumulate around residual tumor defects and promote immunosuppression, recurrence, and metastasis [17]. Notably, recurrent osteosarcoma tissues are characterized by a high proportion of M2‐polarized TAMs, which contribute to immunosuppression and tumor progression [18, 19]. In addition, recurrent osteosarcoma tissues frequently exhibit elevated expression of cyclooxygenase‐2 (COX‐2) within TAM‐rich regions [20]. COX‐2 is a key regulator of the inflammatory tumor microenvironment and has been implicated in tumor initiation, progression, metastasis, and resistance to radiotherapy across multiple cancers [21, 22, 23, 24]. Prostaglandin E2 (PGE_2_), the principal downstream product of COX‐2 signaling, can induce dysfunction of conventional dendritic cells (cDCs), impairing their ability to orchestrate CD8^+^ T‐cell‐mediated antitumor responses and thereby facilitating tumor immune escape [25, 26, 27, 28]. These findings suggest that targeting the COX‐2/PGE_2_ axis within the bone tumor microenvironment represents a promising immunotherapeutic strategy. We therefore hypothesize that simultaneous inhibition of COX‐2 signaling and enhancement of osteosarcoma radiosensitivity may reverse TAM‐mediated immunosuppression and improve the efficacy of radioimmunotherapy.

**FIGURE 1:**
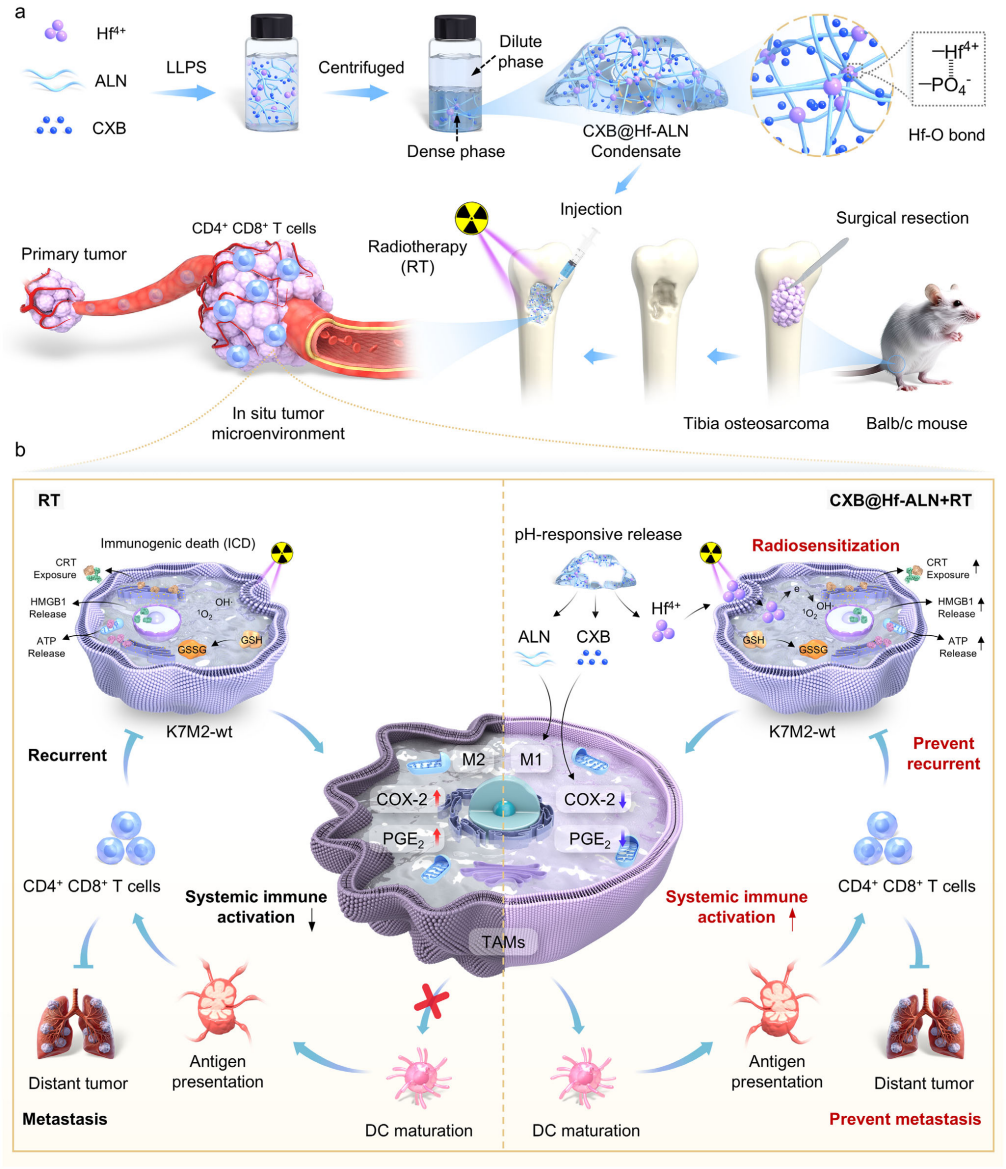
Schematic illustration. (a) The condensate CXB@Hf‐ALN was fabricated via liquid‐liquid phase separation (LLPS) from a mixture of hafnium ions (Hf^4+^), alendronate (ALN), and celecoxib (CXB), and was applied for radiosensitization in post‐operative treatment of orthotopic OS. (b) Under radiotherapy conditions, CXB@Hf‐ALN effectively induced ICD in K7M2‐wt cells. Furthermore, it promoted macrophage M1 polarization and inhibited COX‐2/PGE_2_ expression, ultimately leading to significant suppression of primary OS growth and distant lung metastasis.

Surgical debulking remains the primary clinical intervention for osteosarcoma, and the postoperative bone defect provides a valuable opportunity for localized drug delivery [29]. Concentrating radiosensitizers and immunomodulatory agents within the resection cavity could enable precise elimination of residual tumor cells while minimizing systemic toxicity [30]. Among emerging delivery platforms, biomolecular condensates have attracted increasing attention due to their excellent biocompatibility, high drug‐loading capacity for diverse cargos, and low viscosity that enables efficient filling of irregular defects [31, 32, 33]. Here, to eliminate residual tumor cells and prevent postoperative recurrence, we develop an injectable metal‐drug supramolecular condensate by exploiting liquid‐liquid phase separation (LLPS) driven by coordination interactions between Hf^4+^ ions and the phosphonate groups of alendronate (ALN). This Hf‐ALN condensate was further used to deliver the COX‐2 inhibitor celecoxib (CXB). After intracavitary injection, Hf‐mediated radiosensitization enhances X‐ray energy deposition and reactive oxygen species (ROS) generation, thereby amplifying immunogenic cell death (ICD) in residual tumor cells (Figure 1a). Meanwhile, the acidic tumor microenvironment triggers sustained release of ALN and CXB. ALN promotes the repolarization of M2‐like TAMs toward an M1‐like pro‐inflammatory phenotype, whereas CXB inhibits the COX‐2/PGE_2_ pathway to restore dendritic cell maturation and antigen presentation (Figure 1b). Together, these effects reduce regulatory T‐cell infiltration, enhance CD8^+^ T‐cell–mediated antitumor immunity, eliminate postoperative residual tumor cells, and potentiate systemic antitumor responses consistent with systemic immune activation, ultimately suppressing metastatic tumor growth.

## 2 Results and Discussion

### 2.1 COX‐2 Upregulation and M2 Macrophage Enrichment in Recurrent Osteosarcoma

**FIGURE 2:**
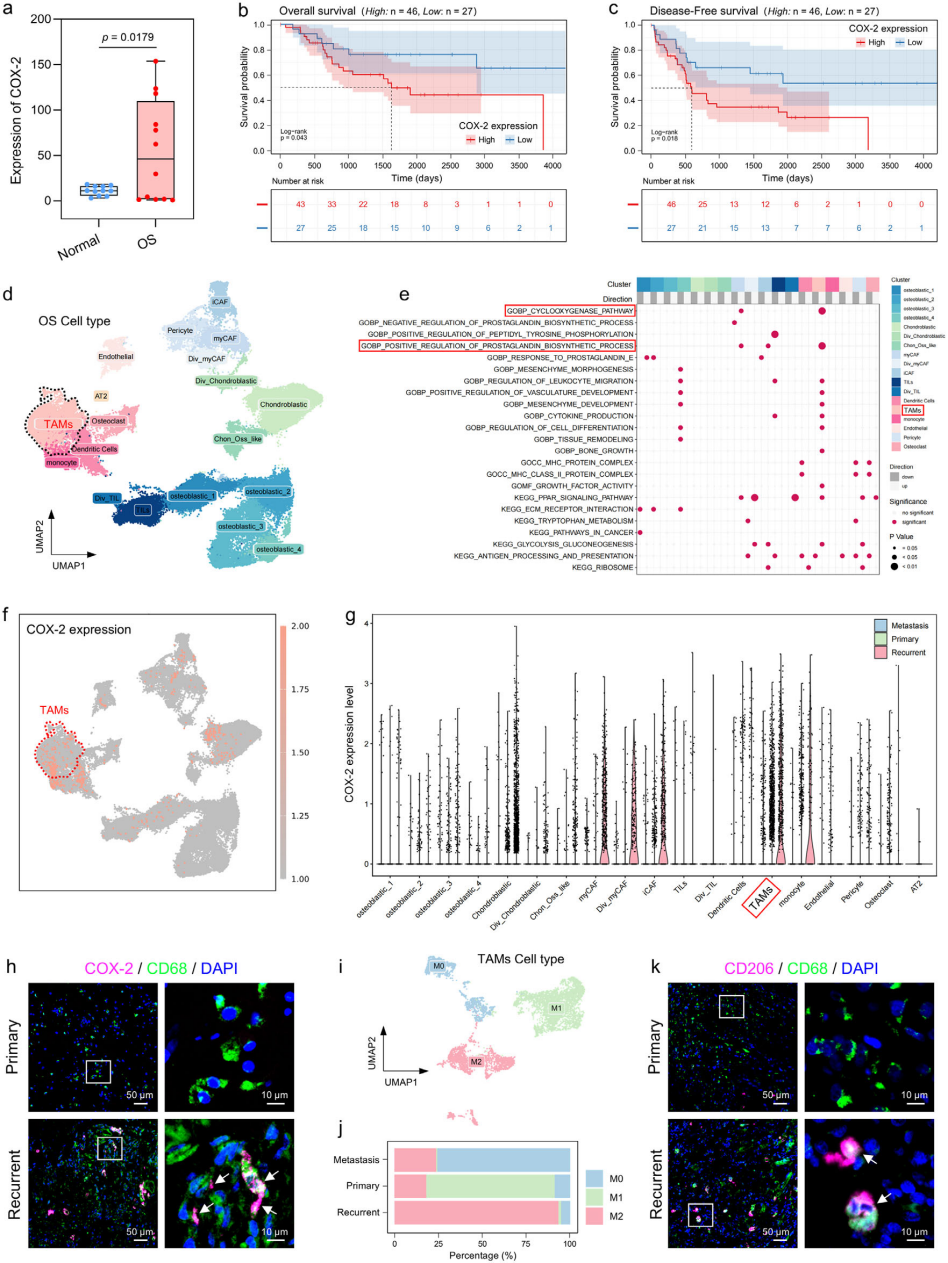
Patients with recurrent osteosarcoma exhibit up‐regulated COX‐2 expression and a reduced survival period. (a) Box plot of COX‐2 expression in healthy individuals (*n* = 11) and osteosarcoma patients (*n* = 12). (b) Survival curve of osteosarcoma patients with high COX‐2 expression and low COX‐2 expression. (c) Disease‐Free survival curve of osteosarcoma patients with high COX‐2 expression and low COX‐2 expression. (d) UMAP visualization of the 17 cellular clusters identified in OS tissues. (e) Heatmap of GSEA results for the 17 cellular clusters in primary and recurrent OS, showing 21 significantly enriched GO and KEGG pathways. (f) UMAP projection illustrating the expression levels of the COX‐2 gene in OS tissues. (g) Analysis of differential COX‐2 expression across various OS tissue types and cellular clusters. (h) Immunofluorescence staining showing the expression of COX‐2 and CD68 in primary and recurrent OS tissues, the white arrow indicates the macrophages expressing COX‐2, *n* = 5. (i) UMAP visualization of different cell types of TAMs in OS tissue. (j) Relative proportions of different cell types within the TAMs population in different OS samples (primary, recurrent, and metastasis). (k) Immunofluorescence staining showing the expression of CD206 and CD68 in primary and recurrent OS tissues, the white arrow indicates the macrophages expressing CD206, *n* = 5. Data are presented as mean values ± SEM. The significance of differences was assessed using a two‐sided Student's *t*‐test with Tukey's multiple comparison test.

To explore the potential role of COX‐2 in osteosarcoma progression and recurrence, bioinformatics analysis was first performed using data from Gene Expression Omnibus (GEO) database and The Cancer Genome Atlas (TCGA). Gene expression analysis based on the GSE126209 dataset revealed that COX‐2 expression was significantly up‐regulated in osteosarcoma patients (*n* = 12) compared to healthy individuals (*n* = 11) (Figure 2a). Based on analyses of overall survival, disease‐free survival (DFS), recurrence‐free survival (RFS), and progression‐free survival (PFS) using the TCGA database, high COX‐2 expression was consistently associated with poor prognosis in osteosarcoma patients. During a 192‐month follow‐up period, the overall survival rate of the high COX‐2 expression group remained consistently lower than that of the low COX‐2 expression group (Figure 2b,c and Figure S1a,b and Table S1), suggesting that elevated COX‐2 expression is negatively correlated with patient survival and may serve as a prognostic biomarker for osteosarcoma.

To further investigate the cellular sources and functional significance of COX‐2 expression, we analyzed the single‐cell transcriptomic dataset GSE152048, which includes seven osteosarcoma samples comprising three primary (BC2, BC16, BC22), two metastatic (BC10, BC17), and two recurrent cases (BC11, BC20). Single‐cell analysis revealed that osteosarcoma tissues form a highly heterogeneous cellular ecosystem, with 17 distinct cell subpopulations identified (Figure 2d). Comparative analysis demonstrated substantial differences in the composition (Figure S1c) and relative abundance (Figure S1d) of these cell subpopulations between primary and recurrent osteosarcoma tissues, suggesting that specific cellular populations may contribute to tumor progression and recurrence. Gene Set Enrichment Analysis (GSEA) further revealed functional specialization among these cell clusters (Figure 2e). Cell‐cycle‐related pathways were predominantly enriched in highly proliferative tumor cell clusters, whereas immune‐related pathways were mainly enriched in T cells and macrophage populations. Notably, macrophage clusters associated with recurrent osteosarcoma showed significant activation of the cyclooxygenase pathway and the positive regulation of prostaglandin biosynthetic processes, indicating a potential role of inflammatory signaling in disease progression. Further examination of the inflammation‐related gene COX‐2 revealed strong cell‐type specificity, with the highest expression observed in macrophage subpopulations (Figure 2f). Importantly, the overall expression level of COX‐2 was significantly elevated in recurrent osteosarcoma tissues compared with primary samples (Figure 2g), suggesting a close association with tumor recurrence and poor clinical outcomes. To validate these findings at the tissue level, immunohistochemical (IHC) and immunofluorescence (IF) analyses were performed on paired clinical samples from five patients with primary osteosarcoma and their corresponding recurrent lesions (Figure S1e). Consistent with the transcriptomic results, recurrent tumor tissues exhibited markedly higher COX‐2 expression than primary tumors. Moreover, at the position indicated by the white arrow, COX‐2 was colocalized with TAMs labeled by CD68 in recurrent tumor samples (Figure 2h). These results further support the association between COX‐2 upregulation in TAMs and OS progression.

Next, we performed clustering analysis of immune cell populations in OS tissues. The immune cell populations mainly consisted of TAMs, neutrophils, B cells, dendritic cells (DCs), T cells, natural killer (NK) cells, osteoclasts, and mast cells (Figure S1f). Among these, TAMs were the predominant immune cell type in the OS tumor microenvironment, accounting for more than 50% of infiltrating immune cells (Figure S1g). Further clustering analysis of the TAM population revealed that TAMs were primarily composed of unpolarized macrophages (M0), M1‐like macrophages, and M2‐like macrophages (Figure 2i). Of note, recurrent osteosarcoma tissues exhibited a higher proportion of M2‐like TAMs, which are known to contribute to immunosuppression and tumor progression (Figure 2j). This result was further validated in clinical tumor samples (Figure 2k). Collectively, these findings demonstrate that COX‐2 upregulation and M2 TAM enrichment are key features of osteosarcoma recurrence. Targeting COX‐2 or modulating TAM polarization may represent a potential therapeutic strategy to prevent osteosarcoma recurrence.

### 2.2 Synthesis and Characterization of the Condensate System

To evaluate the feasibility of condensate formation, computational simulations were first performed to investigate the coordination interactions between alendronate (ALN) and Hf^4+^ ions. Density functional theory (DFT) geometry optimization was used to calculate the electrostatic potential surface (EPS) distribution of ALN (Figure S2a). The phosphate, hydroxyl, and amino groups displayed strong negative electrostatic potentials, indicating their ability to coordinate with positively charged Hf^4+^ ions. Structural modeling further suggested that the phosphate groups of ALN form stable coordination bonds with Hf^4+^ (Figure S2b).

**FIGURE 3:**
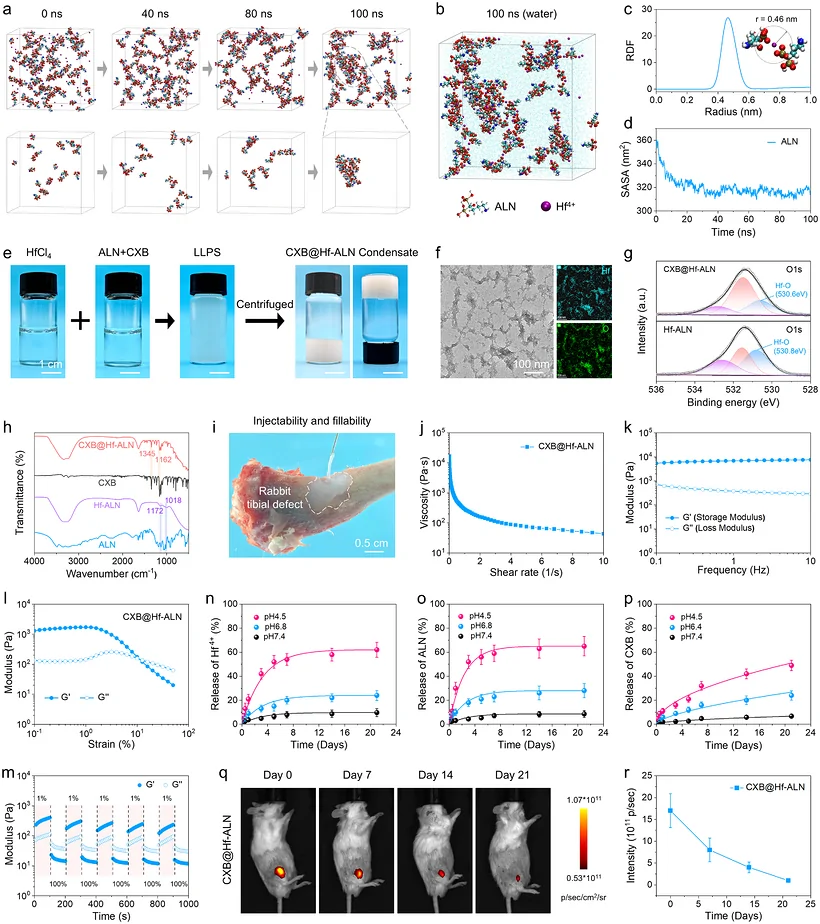
Simulation of Hf‐ALN and preparation and characterization of CXB@Hf‐ALN. (a, b) Molecular dynamics simulation of the mixed system of HfCl_4_ aqueous solution and ALN aqueous solution over 0–100 ns. (c) Radial distribution function (RDF) analysis of the mixed system. (d) Solvent accessible surface area (SASA) analysis of the mixed system. (e) Photograph of CXB@Hf‐ALN condensate obtained after mixing and stirring HfCl_4_ aqueous solution with ALN aqueous solution containing CXB, followed by centrifugation. (f) TEM image and EDS mapping images of CXB@Hf‐ALN. (g) High‐resolution XPS O1s spectra of Hf‐ALN and CXB@Hf‐ALN. (h) FTIR spectra of ALN, CXB, Hf‐ALN, and CXB@Hf‐ALN. (i) Photographs demonstrating the injectability of CXB@Hf‐ALN and its filling of a Ex vivo rabbit tibial bone defect site. (j) Shear‐thinning behavior of the CXB@Hf‐ALN. (k) Stability evaluation of the CXB@Hf‐ALN. (l) Strain amplitude sweep of the CXB@Hf‐ALN. (m) Modulus changes of the CXB@Hf‐ALN under alternating cyclic strains of 1% and 100%. (n–p) Acid‐responsive release profiles of (n) Hf^4+^, (o) ALN, and (p) CXB from the CXB@Hf‐ALN. (q) Representative fluorescence micrographs of BALB/c mice at different time points after intramuscular injection of ICG‐labeled CXB@Hf‐ALN (*n* = 5). (r) Fluorescence intensity at different time points. Data are presented as mean values ± SEM.

To further explore the assembly behavior, molecular dynamics (MD) simulations were conducted to monitor the conformational evolution of the ALN‐Hf system over 0–100 ns. The simulation box reached an equilibrium size of approximately 8 × 8 × 8 nm^3^, and the trajectories revealed pronounced aggregation between ALN molecules and Hf^4+^ ions during the simulation (Figure 3a,b). Binding energy analysis showed that the interaction energy gradually increased and stabilized after approximately 80 ns, suggesting the formation of stable coordination complexes (Figure S2c). In MD simulations, non‐bonded interactions include van der Waals forces described by the Lennard‐Jones potential and electrostatic interactions described by Coulomb's law. The negative binding energy observed here indicates strong attractive interactions between ALN and Hf^4+^ ions.

The structural organization of the assembled system was further analyzed using the radial distribution function (RDF). The RDF curve exhibited a prominent first peak at 0.46 nm, corresponding to the average nearest‐neighbor distance and indicating short‐range ordered structures within the assembly (Figure 3c). Integration of the RDF curve provided the coordination number as a function of distance (Figure S2d). In addition, the solvent‐accessible surface area (SASA) decreased progressively during the simulation (Figure 3d), reflecting increased molecular aggregation and compaction of the assembly. Together, these simulations demonstrate that ALN can effectively coordinate with Hf^4+^ ions to form stable supramolecular complexes, providing theoretical support for the formation of the Hf‐ALN condensate system.

To validate these predictions experimentally, the formation and physicochemical properties of the Hf‐ALN condensate were investigated. Macroscopic observation revealed that mixing Hf^4^^+^ and ALN solutions induced liquid‐liquid phase separation, resulting in a white turbid solution. After centrifugation and removal of the supernatant, an Hf‐ALN condensate was obtained (Figure S3). Using the same procedure in the presence of celecoxib (CXB) yielded the CXB@Hf‐ALN condensate (Figure 3e). Laser diffraction particle size analysis showed that the mixed solution initially exhibited an average particle size of approximately 118 nm, while after 3 h of reaction a new peak emerged at approximately 30 nm, indicating dynamic structural evolution during the assembly process (Figure S4).

Transmission electron microscopy (TEM) revealed that the injectable CXB@Hf‐ALN condensate possessed a porous network structure, while elemental mapping confirmed homogeneous distribution of Hf and O throughout the framework (Figure 3f). X‐ray photoelectron spectroscopy (XPS) analysis of the O1s spectrum showed a lattice oxygen peak at 530.8 eV, corresponding to Hf‐O coordination bonds formed between Hf^4^^+^ and ALN (Figure 3g). In addition, sulfur (S) and fluorine (F) signals corresponding to 1.43% and 2.21%, respectively, were detected in CXB@Hf‐ALN, confirming successful incorporation of celecoxib (Figure S5a–f). Fourier‐transform infrared (FTIR) spectroscopy further supported this conclusion: characteristic phosphate peaks of ALN appeared at 1172 cm^−1^ with P─O stretching vibrations at 1018 cm^−1^, while additional peaks at 1345 and 1162 cm^−1^ corresponding to the sulfonyl groups of celecoxib were observed in CXB@Hf‐ALN (Figure 3h). Circular dichroism (CD) spectroscopy revealed a negative peak at 190–200 nm, characteristic of a random coil conformation, and another peak near 300 nm, indicating the presence of chiral supramolecular structures (Figure S6a). X‐ray diffraction (XRD) patterns further showed that while HfCl_4_ and ALN exhibited strong crystallinity, the assembled condensate displayed an amorphous structure, consistent with supramolecular assembly via coordination interactions (Figure S6b). For CXB loading, the content of CXB in the supernatant of the CXB@Hf‐ALN sample after centrifugation was determined by HPLC. Subsequently, through formula calculation, the encapsulation efficiency and drug‐loading content were 96.31%±1.65% and 1.83%±0.27%, respectively (Figure S7a,b).

The rheological properties of the condensates were subsequently evaluated. As shown in Figure 3i and Movie S1, CXB@Hf‐ALN exhibited excellent injectability, allowing it to fully fill irregular bone defects in an ex vivo rabbit tibia model. Viscosity‐shear rate measurements revealed pronounced shear‐thinning behavior, with viscosity decreasing as shear rate increased (Figure 3j and Figure S8a), indicating favorable injectability. Frequency sweep analysis demonstrated that the storage modulus (G′) was significantly higher than the loss modulus (G″), confirming the robust structural stability of the condensate network (Figure 3k and Figure S8b). Alternating strain cycle tests further revealed rapid and reversible sol‐gel transitions, indicating excellent self‐healing properties of both condensates (Figure 3l,m and Figure S8c,d). Notably, the rheological properties of Hf‐ALN and CXB@Hf‐ALN were comparable, suggesting that celecoxib loading does not significantly alter the mechanical characteristics of the condensate system.

The release profiles of Hf^4^^+^, ALN, and CXB from the CXB@Hf‐ALN condensate were then evaluated under different pH conditions over 0.25‐21 days. At physiological pH (7.4), release in PBS was relatively slow, whereas it accelerated markedly under mildly acidic conditions (pH 6.8 and 5.0), demonstrating pH‐responsive release behavior (Figure 3n–p). The release profile of ALN closely paralleled that of Hf^4+^, and nearly complete release of both species was observed at pH 4.5. As the Hf‐ALN condensate gradually degraded, encapsulated celecoxib was released in a sustained manner.

In addition, the in vivo degradation and retention of the condensates were evaluated using indocyanine green (ICG)‐labeled materials injected into the hind limb muscles of BALB/c mice. Fluorescence imaging showed that signal intensity gradually decreased over time (Day 7, Day 14, and Day 21), indicating progressive degradation while maintaining localized retention for more than three weeks (Figure 3q,r and Figure S9a,b). Collectively, these results confirm the successful construction of a stable, injectable, and pH‐responsive Hf‐ALN condensate system capable of sustained drug release and long‐term local retention, providing a solid physicochemical foundation for subsequent radioimmunotherapeutic applications.

### 2.3 Radiosensitization Effect of the CXB@Hf‐ALN Condensate

**FIGURE 4:**
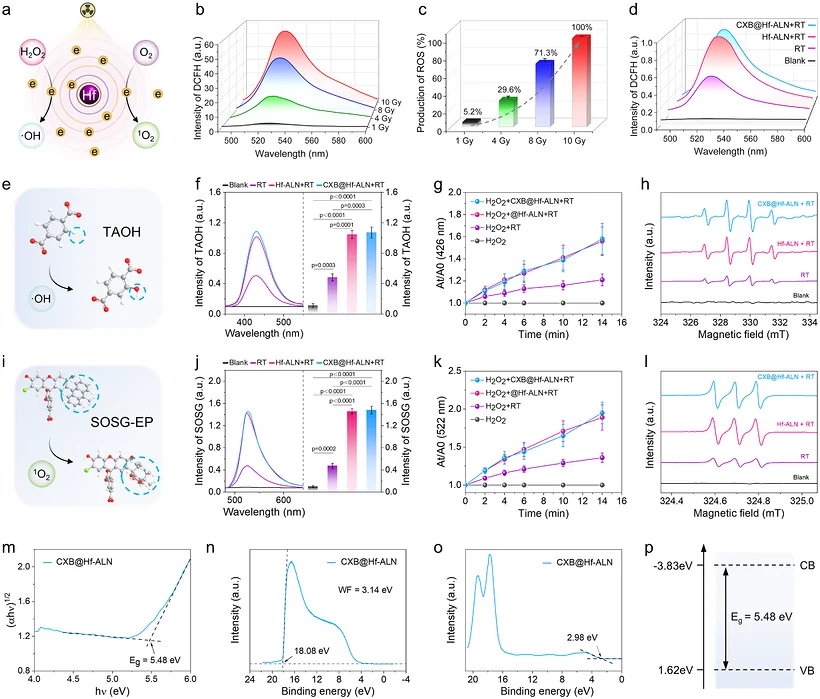
Radiosensitizing Effect of the CXB@Hf‐ALN. (a) Schematic illustration of ROS generation by hafnium upon X‐ray irradiation. (b, c) Total ROS production curves (b) and corresponding quantification (c) under different radiation doses, measured using the DCFH probe. (d) Total ROS generation curves under 8 Gy irradiation for different treatment groups. (e) Reaction scheme of disodium terephthalate (TA) with •OH to form hydroxyterephthalic acid (TAOH). (f) Fluorescence spectra and quantitative analysis of TAOH, reflecting •OH generation across different groups. (g) Time‐dependent fluorescence changes of the TA probe under various treatments. (h) EPR spectra of •OH trapped by DMPO in different conditions. (i) Reaction scheme of SOSG with ^1^O_2_ to form SOSG endoperoxide (SOSG‐EP). (j) Fluorescence spectra and quantitative analysis of SOSG‐EP, indicating ^1^O_2_ production in each group. (k) Time‐dependent fluorescence changes of the SOSG probe under different treatments. (l) EPR spectra of ^1^O_2_ trapped by TEMP under various conditions. (m) Bandgap energy of the CXB@Hf‐ALN. (n) UPS spectrum and work function of the CXB@Hf‐ALN. (o) XPS valence band spectrum of the CXB@Hf‐ALN. (p) Energy level diagram illustrating the Fermi level of the CXB@Hf‐ALN. Data are presented as mean values ± SEM. The significance of differences was assessed using a two‐sided Student's *t*‐test with Tukey's multiple comparison test.

Given the well‐established radiosensitizing capability of hafnium‐based materials, we next investigated whether the CXB@Hf‐ALN condensate could enhance ROS generation under X‐ray irradiation. As illustrated in Figure 4a, the proposed radiosensitization mechanism involves irradiation‐induced electron‐hole generation in the Hf‐containing condensate, which subsequently catalyzes ROS production. The overall ROS generation was first evaluated using the fluorescent probe DCFH‐DA. As shown in Figure 4b,c, ROS levels increased significantly with increasing radiation dose (1, 4, 8, and 10 Gy), confirming the strong radiosensitizing capability of the material. Notably, the combination of CXB@Hf‐ALN and RT (8 Gy) produced the highest ROS levels among all treatment groups (Figure 4d). To further identify the specific ROS species involved, hydroxyl radical (•OH) production was examined using disodium terephthalate (TA) as a fluorescent probe. TA reacts with •OH to generate the fluorescent product 2‐hydroxyterephthalic acid (TAOH), whose fluorescence intensity correlates with •OH concentration (Figure 4e). Minimal •OH generation was detected in the blank group, whereas the RT group showed a noticeable increase. Both Hf‐ALN+RT and CXB@Hf‐ALN+RT groups exhibited significantly higher •OH production, with no statistical difference between them (Figure 4f). Under combined treatment with CXB@Hf‐ALN and RT, the absorbance of TAOH gradually decreased over a 14‐min irradiation period, indicating continuous radical generation during irradiation (Figure 4g).

Electron spin resonance (ESR) spectroscopy further confirmed the formation of hydroxyl radicals [34, 35]. A characteristic 1:2:2:1 quartet ESR signal corresponding to •OH was detected in the RT, Hf‐ALN+RT, and CXB@Hf‐ALN+RT groups (Figure 4h), whereas no signal was observed in the blank control. Compared with RT alone, both Hf‐ALN+RT and CXB@Hf‐ALN+RT groups displayed markedly stronger ESR signals, indicating enhanced radical generation mediated by the Hf‐based condensate. In addition to hydroxyl radicals, intracellular singlet oxygen (^1^O_2_) generation was evaluated using the singlet oxygen sensor green (SOSG) assay (Figure 4i). Both Hf‐ALN+RT and CXB@Hf‐ALN+RT groups produced significantly higher levels of ^1^O_2_ compared with RT alone (Figure 4j). Consistently, SOSG absorbance gradually increased over time under CXB@Hf‐ALN+RT treatment (Figure 4k), indicating sustained ROS production during irradiation. ESR spectroscopy further verified the formation of ^1^O_2_, as evidenced by a characteristic 1:1:1 triplet signal detected in the RT, Hf‐ALN+RT, and CXB@Hf‐ALN+RT groups (Figure 4l), while no signal was observed in the blank group. These results suggest that the enhanced radiosensitization effect is primarily attributable to the catalytic activity of the Hf component within the condensate.

To further elucidate the mechanism underlying ROS generation, the electronic band structure of CXB@Hf‐ALN was investigated. The optical bandgap was determined using solid‐state UV–vis absorption spectroscopy combined with Kubelka‐Munk transformation, yielding a bandgap value of 5.48 eV (Figure 4m). In addition, Fermi level analysis estimated the work function of CXB@Hf‐ALN to be 3.14 eV (Figure 4n). X‐ray photoelectron spectroscopy revealed that the valence band edge relative to the vacuum level was located at 2.98 eV (Figure 4o). Based on these results, the valence band maximum (EVB) and conduction band minimum (ECB) relative to the normal hydrogen electrode (NHE) were calculated to be 1.62 and −3.83 eV, respectively (Figure 4p).

According to this band structure, X‐ray irradiation induces electron‐hole pair formation in the CXB@Hf‐ALN condensate. The excited electrons reduce dissolved O_2_ to superoxide radicals (O_2_^−^), which subsequently convert into ^1^O_2_, while the generated holes can activate H_2_O_2_ to produce •OH radicals. Together, these ROS species contribute to enhanced oxidative stress and tumor cell damage under radiotherapy (Figure 4a). Collectively, these results demonstrate that CXB@Hf‐ALN effectively amplifies radiation‐induced ROS generation, providing a mechanistic basis for its potent radiosensitization capability.

### 2.4 Radiosensitization of CXB@Hf‐ALN Condensate Promotes Immunogenic Cell Death In Vitro

**FIGURE 5:**
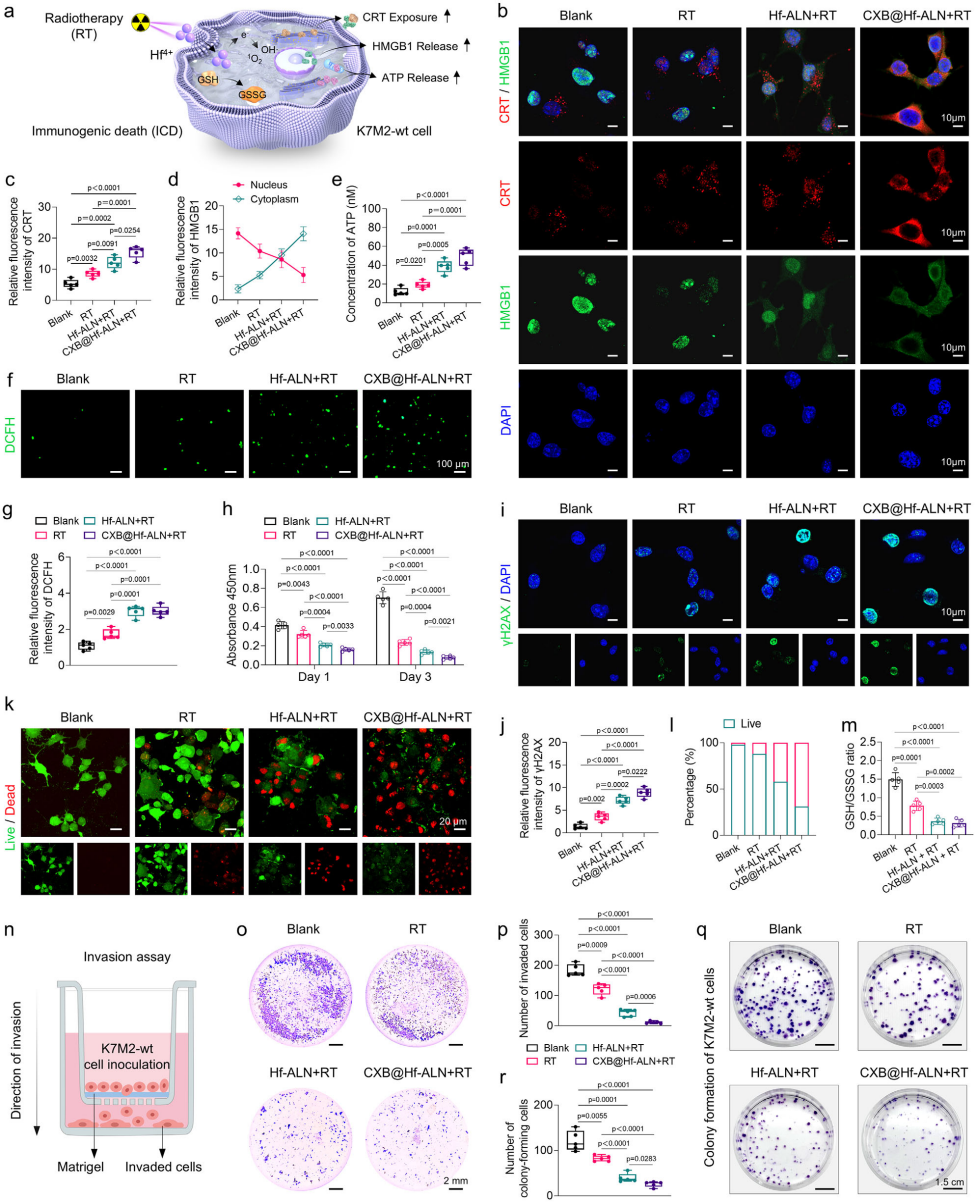
CXB@Hf‐ALN+RT Enhances Immunogenic Cell Death in K7M2‐wt Cells. (a) Schematic diagram illustrating the ICD mechanism in K7M2‐wt cells. (b) Representative immunofluorescence images of ICD markers (CRT and HMGB1) in K7M2‐wt cells treated with CXB@Hf‐ALN+RT, *n* = 5. (c) Quantification of CRT fluorescence intensity. (d) Quantification of HMGB1 fluorescence intensity. (e) ATP concentration in the supernatant of K7M2‐wt cells after CXB@Hf‐ALN+RT treatment. (f) Representative images of ROS staining in treated K7M2‐wt cells, *n* = 5. (g) Quantitative analysis of ROS fluorescence intensity. (h) Cell viability of K7M2‐wt cells following CXB@Hf‐ALN+RT treatment, as determined by CCK‐8 assay. (i) Representative immunofluorescence images of the DNA damage marker γ‐H2AX in treated cells, *n* = 5. (j) Quantification of γ‐H2AX fluorescence intensity. (k) Representative live/dead staining images (calcein‐AM for live cells, propidium iodide for dead cells) of K7M2‐wt cells after treatment, *n* = 5. (l) Ratio of live to dead cells under CXB@Hf‐ALN+RT treatment. (m) Ratio of reduced glutathione (GSH) to oxidized glutathione (GSSG) in K7M2‐wt cells after CXB@Hf‐ALN+RT treatment. (n) Schematic diagram of the invasion assay. (o) Anti‐invasive effects of different sample treatments on K7M2‐wt cells, *n* = 5. (p) Number of invaded cells. (q) Colony formation of K7M2‐wt cells under different sample treatments, *n* = 5. (r) Number of colony‐forming cells. Data are presented as mean values ± SEM. The significance of differences was assessed using a two‐sided Student's *t*‐test with Tukey's multiple comparison test.

To investigate whether the CXB@Hf‐ALN condensate enhances ICD under radiotherapy, three hallmark ICD‐associated biomarkers—calreticulin (CRT), high mobility group box 1 (HMGB1), and adenosine triphosphate (ATP)—were examined in K7M2‐wt osteosarcoma cells [36, 37] (Figure 5a). Immunofluorescence staining showed that treatment with CXB@Hf‐ALN combined with RT markedly increased CRT exposure on the cell membrane and promoted HMGB1 translocation from the nucleus to the cytoplasm (Figure 5b–d). In contrast, only limited HMGB1 redistribution was observed in the RT‐alone and Hf‐ALN+RT groups. In addition, ATP release from K7M2‐wt cells was significantly elevated following combined CXB@Hf‐ALN and RT treatment (Figure 5e). These results indicate that CXB@Hf‐ALN synergizes with RT to induce a stronger ICD response.

Given that ROS play a key role in radiation‐induced ICD, intracellular ROS generation was measured using the fluorescent probe DCFH‐DA. Compared with the blank control, RT alone produced noticeable ROS signals, whereas cells treated with condensate‐based therapies (Hf‐ALN+RT and CXB@Hf‐ALN+RT) exhibited substantially stronger fluorescence signals (Figure 5f,g). These results suggest that the Hf component of the condensate significantly amplifies ROS production under irradiation. The cytotoxic effects of the treatments were evaluated using a CCK‐8 assay, which showed that condensate‐assisted RT significantly reduced K7M2‐wt cell viability compared with RT alone (Figure 5h). To clarify whether ROS generation contributes to the enhanced antitumor efficacy of CXB@Hf‐ALN+RT, N‐acetyl‐L‐cysteine (NAC) was introduced as a ROS scavenger for mechanistic validation. DCFH‐DA staining demonstrated that CXB@Hf‐ALN+RT markedly increased intracellular ROS accumulation compared with individual treatments, whereas NAC treatment effectively eliminated the excessive ROS production (Figure S10a,b). Furthermore, we evaluated whether ROS depletion affected ICD. CXB@Hf‐ALN+RT significantly promoted CRT exposure, extracellular ATP release, and HMGB1 release in K7M2‐wt cells. However, these ICD‐associated effects were substantially attenuated after NAC administration, indicating that ROS generation is essential for CXB@Hf‐ALN+RT‐induced ICD (Figure S10c–f).

DNA double‐strand break formation was further assessed using γ‐H2AX staining, a widely used marker of DNA damage. Immunofluorescence imaging revealed a pronounced increase in γ‐H2AX foci in cells treated with CXB@Hf‐ALN+RT, indicating enhanced radiation‐induced DNA damage (Figure 5i,j). Cell death was further analyzed using Calcein‐AM/propidium iodide (PI) live/dead staining. Compared with the blank control, RT alone induced moderate cell death, whereas treatment with Hf‐ALN+RT and CXB@Hf‐ALN+RT resulted in markedly increased PI‐positive cells (red fluorescence), demonstrating enhanced cytotoxicity (Figure 5k,l). To further examine the oxidative stress induced by the treatment, intracellular levels of reduced glutathione (GSH) and oxidized glutathione (GSSG) were measured. The GSH/GSSG ratio, an indicator of cellular redox balance, decreased significantly in RT‐treated K7M2‐wt cells and was further reduced in the Hf‐ALN+RT and CXB@Hf‐ALN+RT groups (Figure 5m). To assess the impact of different treatment conditions on the invasive capacity of K7M2‐wt cells, we performed Transwell invasion assays (Figure 5n). The experimental results demonstrated that the various treatments significantly influenced the invasive ability of the tumor cells. Notably, CXB@Hf‐ALN markedly suppressed the motility of K7M2‐wt cells, with their invasive capacity being significantly reduced compared to all other groups (Figure 5o,p). The unlimited proliferative capacity of tumor cells represents another hallmark of malignancy. In this study, we evaluated the long‐term effects of various treatments on the survival and proliferation of K7M2‐wt cells through colony formation assays (Figure 5q). By counting the number of colonies formed (Figure 5r), we observed trends highly consistent with the results of the invasion assay. The blank control group exhibited robust clonogenicity, generating a large number of colonies. In contrast, the Hf‐ALN+RT treatment group significantly reduced colony formation, indicating that this combined regimen not only inhibits invasion but also effectively kills tumor cells or suppresses their proliferation, thereby preventing clonogenic growth. Similarly, the CXB@Hf‐ALN+RT treatment group demonstrated the most potent anti‐clonogenic effect, with the lowest number of colonies formed among all treatment groups. These findings further validate the critical role of CXB modification in enhancing anti‐tumor efficacy. This decline indicates a disruption of redox homeostasis and increased oxidative stress in tumor cells. These results demonstrate that the CXB@Hf‐ALN condensate markedly enhances radiotherapy‐induced oxidative stress, DNA damage, and ICD in osteosarcoma cells, thereby enhancing the immune response of the system, strengthen the anti‐tumor effect and inhibit distant metastasis.

### 2.5 CXB@Hf‐ALN+RT Suppresses the COX‐2/PGE_2_ Axis and Promotes M1 Polarization of TAMs

**FIGURE 6:**
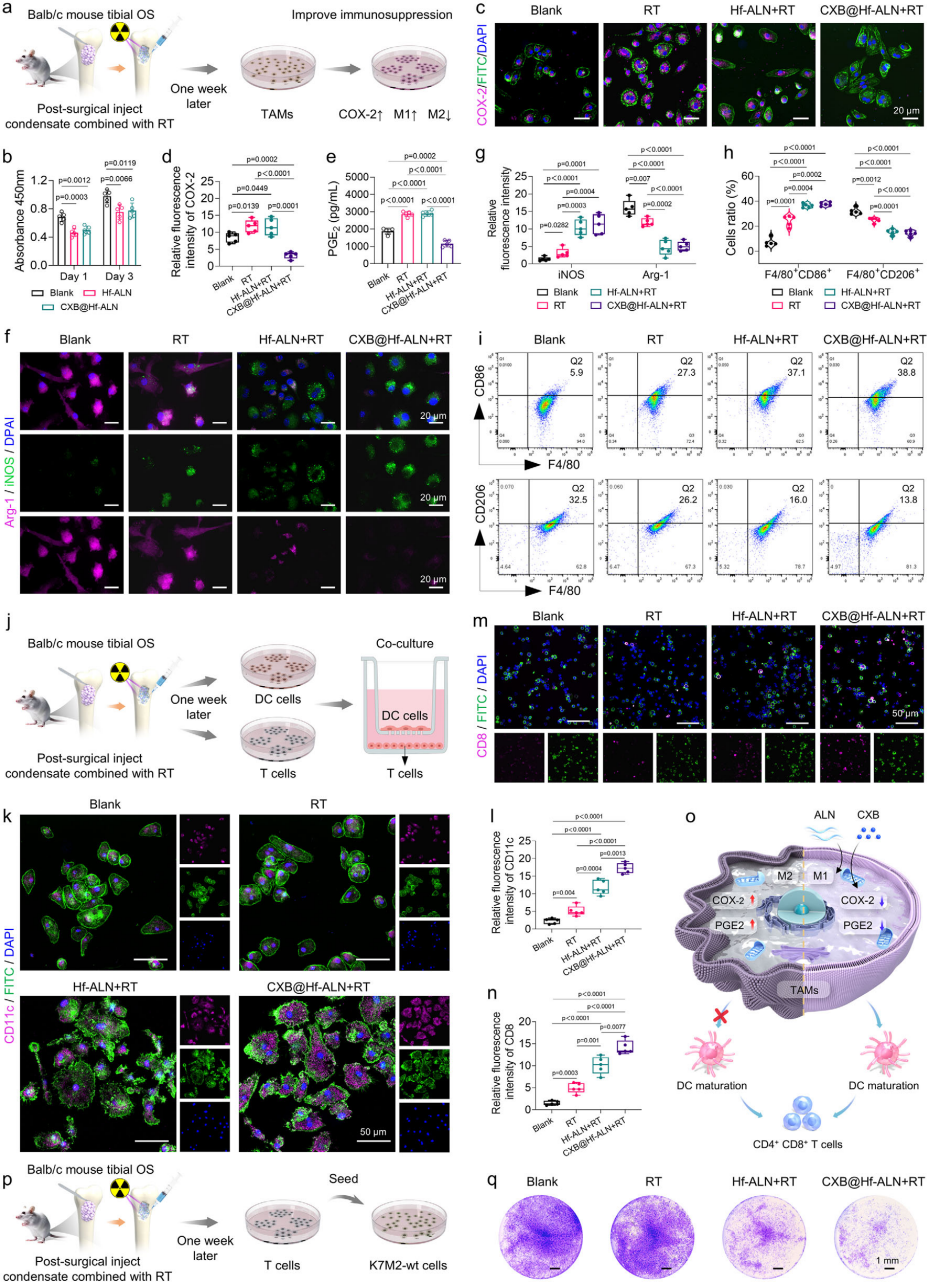
CXB@Hf‐ALN+RT downregulates COX‐2/PGE_2_ and promotes M1 polarization in macrophages. (a) Schematic illustration of the extraction and isolation of macrophages from osteosarcoma tissues. (b) Viability of macrophages treated with CXB@Hf‐ALN, as determined by CCK‐8 assay. (c) Representative immunofluorescence images of COX‐2 expression in macrophages treated with CXB@Hf‐ALN+RT, *n* = 5. (d) Quantitative analysis of COX‐2 fluorescence intensity. (e) PGE_2_ concentration in the supernatant of macrophages after CXB@Hf‐ALN+RT treatment. (f) Representative immunofluorescence images of iNOS and Arg‐1 expression in macrophages treated with CXB@Hf‐ALN, *n* = 5. (g) Quantitative analysis of iNOS and Arg‐1 fluorescence intensity. (h, i) Flow cytometry analysis of F4/80^+^ CD86^+^ and F4/80^+^ CD206^+^ expression in macrophages across treatment groups; *n* = 5. (j) Schematic illustration of the procedure for isolating dendritic cells (DCs) and T cells from the osteosarcoma microenvironment. (k) Representative images showing morphological features and CD11c staining of DCs at the tumor site in osteosarcoma mice after different treatments, *n* = 5. (l) Quantitative analysis of CD11c staining fluorescence intensity. (m) Representative images of CD8 staining in T cells at the tumor site in osteosarcoma mice after different treatments, *n* = 5. (n) Quantitative analysis of CD8 staining fluorescence intensity. (o) Schematic diagram illustrating COX‐2/PGE_2_ pathway downregulation and M1 polarization in macrophages induced by CXB@Hf‐ALN under radiotherapy. (p) Schematic diagram of co‐culture of activated T cells with K7M2‐wt cells. (q) Crystal violet staining of co‐cultured activated T cells and K7M2‐wt cells, *n* = 5. Data are presented as mean values ± SEM. The significance of differences was assessed using a two‐sided Student's *t*‐test with Tukey's multiple comparison test.

To investigate the immunomodulatory effects of CXB@Hf‐ALN on TAMs, macrophages were isolated from recurrent murine osteosarcoma tissues treated under different conditions using F4/80 magnetic bead sorting [38] (Figure 6a). The biocompatibility of the condensate toward macrophages was first evaluated using a CCK‐8 assay. Both Hf‐ALN and CXB@Hf‐ALN exhibited negligible cytotoxicity compared with the blank control, indicating good macrophage compatibility (Figure 6b).

We next examined whether CXB@Hf‐ALN could modulate the COX‐2/PGE_2_ immunosuppressive pathway in TAMs. Immunofluorescence staining with an anti‐COX‐2 antibody revealed strong COX‐2 expression in macrophages isolated from untreated recurrent tumors (Figure 6c,d). Notably, COX‐2 expression was further elevated following radiotherapy alone and in the Hf‐ALN+RT group, indicating that irradiation stimulates COX‐2 upregulation within TAMs. In contrast, macrophages treated with CXB@Hf‐ALN+RT exhibited markedly reduced COX‐2 expression, confirming that CXB effectively inhibits radiation‐induced COX‐2 activation. Because prostaglandin E2 (PGE_2_) is a major immunosuppressive mediator generated downstream of COX‐2, its secretion was further quantified using an Enzyme‐linked immunosorbent assay (ELISA) assay. Macrophages exposed to RT alone displayed significantly elevated PGE_2_ levels, whereas treatment with CXB@Hf‐ALN+RT substantially reduced PGE_2_ production (Figure 6e). These results suggest that CXB‐mediated inhibition of the COX‐2/PGE_2_ axis may help alleviate radiation‐induced immunosuppression and facilitate downstream immune activation, including dendritic cell maturation.

We further evaluated whether the condensate could modulate macrophage polarization. Macrophages were first polarized toward an M2‐like phenotype using theophylline and subsequently treated with the condensates. Immunofluorescence staining revealed that treatment with Hf‐ALN or CXB@Hf‐ALN altered the expression of macrophage polarization markers. Specifically, increased iNOS (M1 marker) expression and reduced Arg‐1 (M2 marker) expression were observed following condensate treatment, indicating repolarization of macrophages toward a pro‐inflammatory M1 phenotype (Figure 6f,g). Flow cytometry analysis further confirmed these findings. The proportion of M1‐like macrophages (F4/80^+^CD86^+^) increased significantly after treatment with Hf‐ALN+RT (37.1%) and CXB@Hf‐ALN+RT (38.8%) compared with the blank group (5.9%). Conversely, the M2 macrophage population (F4/80^+^CD206^+^) decreased from 32.5% in the blank group to 16.0% and 13.8% in the Hf‐ALN+RT and CXB@Hf‐ALN+RT groups, respectively (Figure 6h,i). These results demonstrate that ALN effectively promotes TAM repolarization toward an M1 phenotype, thereby shifting the tumor microenvironment toward an immune‐activating state.

In addition, we examined the effect of CXB@Hf‐ALN combined with radiotherapy on osteoclast activity, which plays an important role in osteosarcoma‐associated bone destruction. Tartrate‐resistant acid phosphatase (TRAP) staining revealed the presence of multinucleated osteoclasts in both the blank and RT groups following RANKL stimulation. In contrast, the Hf‐ALN+RT and CXB@Hf‐ALN+RT groups exhibited markedly fewer TRAP‐positive osteoclasts (Figure S11a–c). These results indicate that the ALN released from the condensate effectively suppresses osteoclast activation, potentially contributing to improved bone microenvironment regulation during osteosarcoma therapy.

### 2.6 CXB@Hf‐ALN+RT Promotes Dendritic Cell Maturation and T‐Cell Activation

To further investigate the immunomodulatory effects of CXB@Hf‐ALN combined with radiotherapy, dendritic cells (DCs) and T cells were isolated from recurrent osteosarcoma tissues using CD11c magnetic bead and Pan T Cell Isolation Kit II sorting, respectively (Figure 6j). Immunofluorescence staining was performed to examine the morphology of CD11c^+^ DCs and the infiltration of CD8^+^ T cells within tumor tissues. Distinct differences in the tumor immune microenvironment were observed among the treatment groups. Compared with the other groups, the CXB@Hf‐ALN+RT group exhibited CD11c^+^ DCs with more pronounced dendritic projections and a more mature cellular morphology (Figure 6k,l), indicating enhanced DC maturation. Concurrently, this group displayed markedly increased infiltration of CD8^+^ T cells, as evidenced by significantly higher CD8 fluorescence intensity within the tumor tissues (Figure 6m,n). These findings suggest that CXB@Hf‐ALN combined with radiotherapy effectively promotes T‐cell activation and enhances local antitumor immune responses.

Although radiotherapy alone can induce ICD and promote the release of tumor‐associated antigens, its ability to activate DCs and stimulate T‐cell infiltration is often limited. While Hf‐ALN functions as a radiosensitizer that enhances local radiation‐induced tumor damage, the combination of Hf‐ALN with radiotherapy alone did not substantially improve immune cell activation. In contrast, CXB@Hf‐ALN provides a dual‐functional therapeutic strategy. The Hf‐ALN component amplifies radiotherapy‐induced tumor cell damage, while celecoxib (CXB) exerts immunomodulatory effects by inhibiting the COX‐2/PGE_2_ immunosuppressive pathway within the tumor microenvironment. Suppression of this pathway alleviates immunosuppression, thereby facilitating DC maturation, enhancing antigen presentation, and promoting cytotoxic CD8^+^ T‐cell activation (Figure 6o). Given the close relationship between osteosarcoma and the immune microenvironment, we evaluated the effects of different pretreatments on T cell activity and subsequent killing of K7M2‐wt cells using a co‐culture system (Figure 6p) and crystal violet staining (Figure 6q). The results showed that in the blank control group, a large number of tumor cells survived, exhibiting the deepest staining, which indicated limited T cell‐mediated killing. In the RT group, a slight reduction in tumor cells was observed, suggesting that radiotherapy may modestly enhance immunogenicity or inhibit proliferation. In the Hf‐ALN+RT group, the number of tumor cells was significantly reduced, accompanied by notably lighter staining. This finding indicates that radiosensitizing therapy not only directly kills tumor cells but may also enhance T cell recognition and clearance by inducing ICD or modulating the cellular phenotype. The CXB@Hf‐ALN+RT group exhibited the most potent synergistic killing effect, with tumor cells being almost completely eliminated following co‐culture. Collectively, these results demonstrate that CXB@Hf‐ALN+RT effectively remodels the tumor immune microenvironment by promoting DC maturation and T‐cell activation, thereby amplifying antitumor immunity.

To verify whether the inhibition of ROS affected the downstream immune activation of ICD. Collect the supernatants from the CXB@Hf‐ALN+RT+NAC treatment of K7M2wt cells and continue to process different immune cells (TAMs, CD11c^+^ DCs, and total T cells). Observe the expression ratio of M1/M2 macrophages, the maturation status of DC cells, and the expression of CD8^+^ T cells. The results showed that NAC treatment significantly increased Arg‐1 expression while markedly decreasing iNOS expression, indicating macrophage polarization toward the M2 phenotype. In the presence of NAC, treatment with CXB@Hf‐ALN combined with radiotherapy induced a trend toward macrophage repolarization from the M2 to the M1 phenotype (Figure S12a–c). IF staining of dendritic cells (DCs) showed that NAC treatment markedly reduced or even eliminated the dendritic processes characteristic of mature DC morphology. Following CXB@Hf‐ALN+RT+NAC treatment, a small number of dendritic processes reappeared within the field of view (Figure S12d–f). Finally, CD8^+^ IF staining of total T cells showed that NAC treatment markedly reduced or even abolished CD8^+^ expression. Following CXB@Hf‐ALN+RT+NAC treatment, a detectable level of CD8^+^ expression reappeared within the field of view. The above data suggest that ROS contribute, at least in part, to macrophage repolarization toward the M1‐like phenotype, DC maturation, and T‐cell activation (Figure S12g–i). Overall, these findings confirm that CXB@Hf‐ALN enhances radiotherapy efficacy by amplifying ROS‐mediated oxidative stress, thereby triggering ICD‐associated immune activation.

To verify whether CD8^+^ T cells were involved in the immune‐mediated anti‐tumor activity triggered by CXB@Hf‐ALN+RT. We performed a CD8^+^ T‐cell depletion‐based co‐culture assay. CD8^+^ T cells were isolated using magnetic beads conjugated with an anti‐CD8 antibody and subsequently subjected to repeated stimulation with an anti‐CD3 antibody to induce CD8^+^ T‐cell exhaustion. The exhaustion efficiency was subsequently verified through CD8 staining and quantification. As shown in Figure S13a,b, CD8 expression was markedly reduced following depletion. We additionally examined the expression of the inhibitory receptors PD‐1 and LAG‐3. Their co‐expression was interpreted as an exhaustion‐associated or dysfunctional phenotype rather than definitive evidence of functional T‐cell exhaustion, because inhibitory‐receptor expression alone does not fully define the functional state of CD8^+^ T cells. The experimental results showed high expression of PD‐1 and LAG‐3, with double‐positive staining, confirming that CD8^+^ T cells were effectively depleted from the lymphocyte population (Figure S13c,d). Then, we co‐cultured K7M2wt osteosarcoma cells with either undepleted lymphocytes or CD8^+^ T‐cell‐depleted lymphocytes under otherwise identical treatment conditions. CXB@Hf‐ALN+RT combined with undepleted lymphocytes produced pronounced tumor‐cell killing. In contrast, removal of CD8^+^ T cells significantly attenuated this cytotoxic effect, as demonstrated by an increased number of viable K7M2‐wt cells and reduced tumor‐cell death in the CD8‐depleted group (Figure S13e–g). Thus, depletion of the CD8^+^ T‐cell compartment partially rescued K7M2wt cell survival, providing functional evidence that CD8^+^ T cells contribute substantially to the immune‐mediated antitumor activity elicited by CXB@Hf‐ALN+RT.

These findings are consistent with previous studies demonstrating that radiotherapy can enhance tumor‐antigen presentation, dendritic‐cell activation, and tumor‐specific CD8^+^ T‐cell responses [39, 40]. Moreover, inhibition of the COX‐2/PGE_2_ axis by celecoxib may alleviate tumor‐associated immunosuppression and preserve CD8^+^ T‐cell effector activity. Therefore, our results indicate that CXB@Hf‐ALN+RT has a direct cytotoxic effect on osteosarcoma cells, and at the same time can promote the anti‐tumor immune response mediated by CD8^+^ T cells.

### 2.7 CXB@Hf‐ALN+RT Exhibits Favorable Biosafety and Potent Therapeutic Efficacy in Orthotopic Osteosarcoma

The systemic biosafety of the CXB@Hf‐ALN condensate was first evaluated through blood biochemistry, hematological analysis, and histological examination of major organs. Blood tests showed no significant differences in alanine aminotransferase (ALT), aspartate aminotransferase (AST), total protein (TP), creatinine (CREA), white blood cells (WBC), hemoglobin (HGB), red blood cells (RBC), or platelets (PLT) between the CXB@Hf‐ALN‐treated mice and the blank control group (Figure S14). Consistently, hematoxylin and eosin (H&E) staining of the heart, liver, spleen, lungs, and kidneys revealed no apparent pathological abnormalities or accumulation of degradation products (Figure S15). These results demonstrate the excellent systemic biocompatibility of the CXB@Hf‐ALN condensate.

**FIGURE 7:**
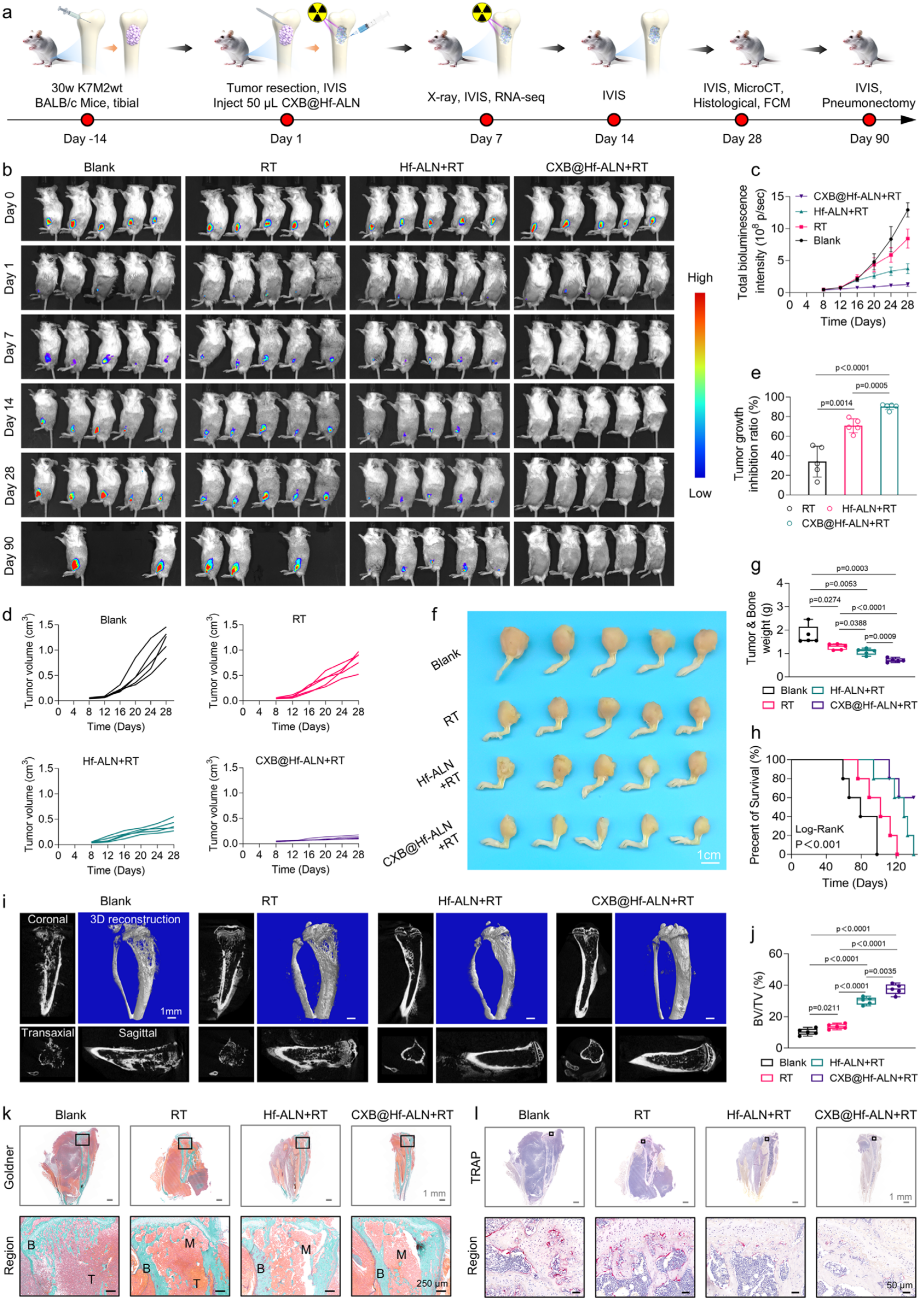
Evaluation of CXB@Hf‐ALN Combined with Radiotherapy in an Orthotopic OS Mouse Model. (a) Schematic diagram of the experimental procedure: establishment of a tibial OS model in Balb/c mice, surgical resection, local injection of CXB@Hf‐ALN and radiotherapy. (b) In vivo bioluminescence imaging (IVIS) of orthotopic OS after treatment, using iRFP720‐expressing K7M2‐wt cells, *n* = 5. (c) Quantitative analysis of bioluminescence intensity. (d) Tumor volume changes over time in different treatment groups. (e) Tumor growth inhibition rate following various treatments. (f) Representative photographs of hind limbs 21 days after orthotopic OS treatment, *n* = 5. (g) Weight of tumors and bone tissues collected from the tibia after 21 days of treatment. (h) Survival curves of Balb/c mice bearing orthotopic OS after different treatments. (i) Representative MicroCT images and 3D reconstructions of tibiae 21 days post‐treatment, *n* = 5. (j) Quantitative analysis of bone volume/total volume (BV/TV) at the tumor site by MicroCT. (k) Representative Goldner's trichrome staining of tibial sections 21 days after treatment (B: bone, M: marrow, T: tumor), *n* = 5. (l) Representative TRAP staining of tibial sections after 21 days of treatment, *n* = 5. Data are presented as mean values ± SEM. The significance of differences was assessed using a two‐sided Student's *t*‐test with Tukey's multiple comparison test.

An orthotopic tibial OS model was established in BALB/c mice using iRFP720 labeled K7M2‐wt cells to evaluate the therapeutic efficacy of the CXB@Hf‐ALN supramolecular condensate combined with radiotherapy (Figure 7a). Tumor progression was monitored in real time by measuring tumor volume, tumor size, and body weight throughout the study. Compared with the Blank group, both RT and Hf‐ALN+RT significantly inhibited OS growth, achieving tumor growth inhibition (TGI) rates of 34.0% and 70.1%, respectively. Notably, treatment with CXB@Hf‐ALN+RT produced a markedly enhanced antitumor effect, reaching a TGI rate of 90.1% (Figure 7b–g). To evaluate long‐term therapeutic outcomes, survival curves were further analyzed. Compared with the Blank group, the combination of radiotherapy and condensate treatment significantly prolonged the survival of tumor‐bearing mice. Among all groups, the CXB@Hf‐ALN+RT group exhibited the most pronounced survival benefit, with a median survival time of 140 days (Figure 7h).

The therapeutic effects on bone destruction were further examined using micro‐computed tomography (MicroCT) and histological analyses. MicroCT images revealed severe bone erosion and incomplete bone morphology in the Blank and RT groups, indicating tumor‐induced bone damage. Although the Hf‐ALN+RT group showed considerable suppression of tumor progression, minor bone erosion remained detectable. In contrast, the CXB@Hf‐ALN+RT group exhibited the most effective tumor suppression with minimal bone destruction (Figure 7i,j and Figure S16a,b). Histological staining further confirmed these findings. Ki67 staining demonstrated a significant reduction in proliferating tumor cells following treatment with Hf‐ALN+RT, and this effect was further enhanced in the CXB@Hf‐ALN+RT group. Meanwhile, TUNEL staining revealed increased apoptosis in tumor tissues treated with CXB@Hf‐ALN+RT compared with RT alone or Hf‐ALN+RT (Figure S17a–c). These results indicate that radiosensitized oxidative stress, together with immune modulation, effectively suppresses tumor cell proliferation and promotes apoptosis. Consistently, γ‐H2AX immunostaining revealed the strongest fluorescence intensity in the CXB@Hf‐ALN+RT group, indicating enhanced radiation‐induced DNA damage within tumor tissues (Figure S17d,e).

Bone remodeling was further evaluated using Goldner's trichrome staining. Extensive bone resorption and severe cortical bone destruction were observed in the Blank group. Although RT alone reduced bone resorption to some extent, residual tumor tissue remained in the bone marrow cavity. In contrast, both the Hf‐ALN+RT and CXB@Hf‐ALN+RT groups exhibited minimal tumor infiltration in the marrow cavity. Notably, the bone structure in the CXB@Hf‐ALN+RT group remained largely intact with negligible bone loss (Figure 7k). In addition, TRAP staining was performed to evaluate osteoclast activity. Numerous TRAP‐positive multinucleated osteoclasts were observed in the Blank and RT groups, whereas the number of osteoclasts was significantly reduced in the Hf‐ALN+RT and CXB@Hf‐ALN+RT groups (Figure 7l). This result indicates that ALN released from the condensate effectively suppresses osteoclast activation, thereby contributing to improved bone preservation. These results demonstrate that CXB@Hf‐ALN combined with radiotherapy effectively suppresses osteosarcoma progression, enhances radiation‐induced DNA damage and tumor apoptosis, preserves bone integrity, inhibits osteoclast activity, and significantly prolongs survival in an orthotopic OS mouse model.

### 2.8 CXB@Hf‐ALN+RT Enhances Immunogenic Cell Death and Activates Systemic Antitumor Immunity

**FIGURE 8:**
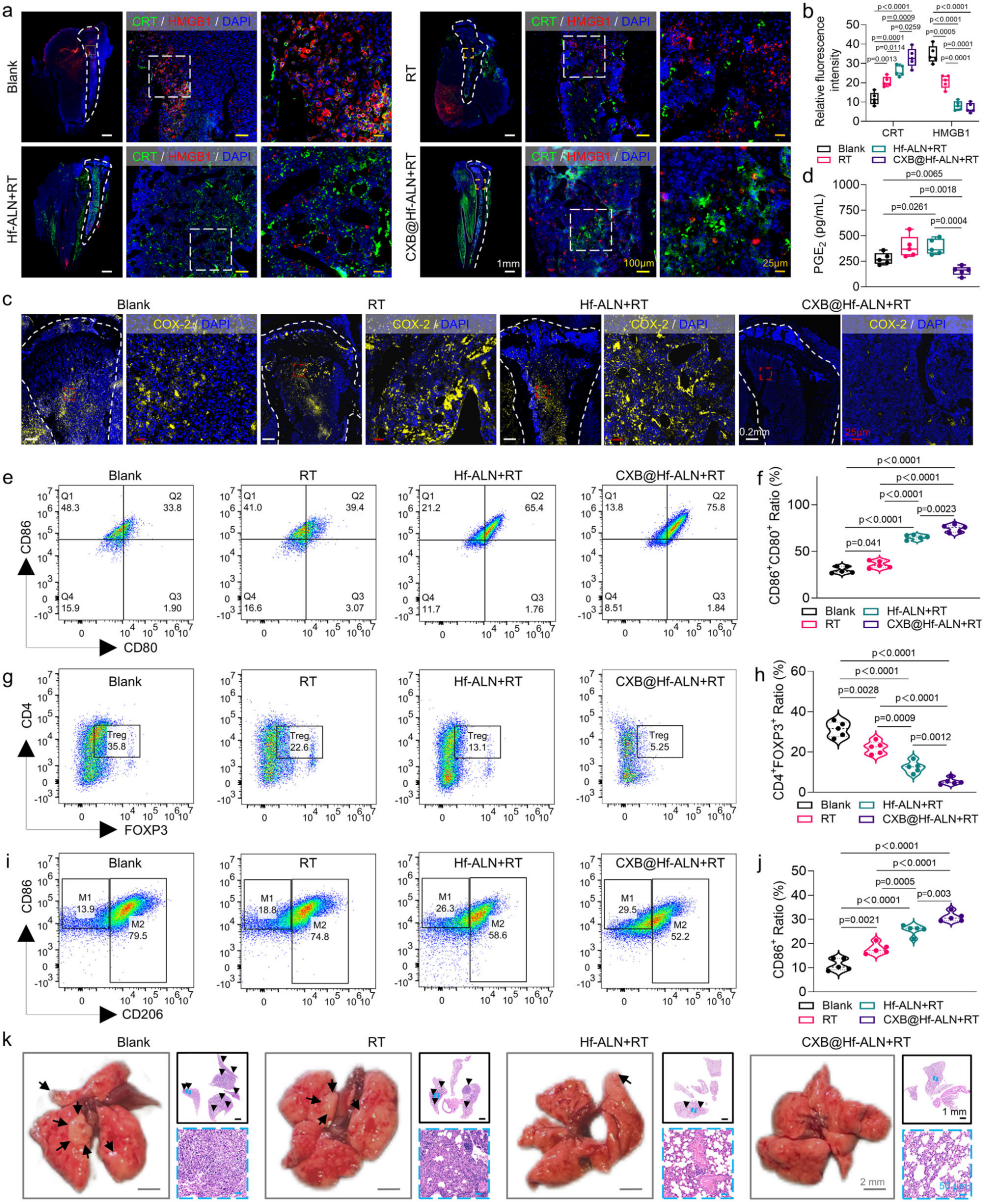
CXB@Hf‐ALN+RT Enhances ICD and Activates Systemic Immunity in Orthotopic OS. (a) Representative immunofluorescence images of co‐staining for CRT (green) and HMGB1 (red) in tibial tumor tissues 21 days after treatment, *n* = 5. (b) Quantitative analysis of CRT and HMGB1 fluorescence intensity. (c) Representative immunofluorescence images of COX‐2 expression in tibial tumor sections after treatment, *n* = 5. (d) Quantitative analysis of PGE_2_ levels in tibial tumor tissues by ELISA 21 days after treatment across different groups. (e, f) Flow cytometry analysis (e) and quantification (f) of CD80^+^CD86^+^ mature dendritic cells infiltrating the tumor, *n* = 5. (g, h) Flow cytometry analysis (g) and quantification (h) of CD4^+^FOXP3^+^ Treg cells within the tumor microenvironment, *n* = 5. (i, j) Flow cytometry analysis (i) and quantification (j) of CD86^+^CD206^+^ macrophages in the tumor tissue, *n* = 5. (k) Representative photographs of lung metastases and corresponding H&E‐stained sections, *n* = 5. Data are presented as mean values ± SEM. The significance of differences was assessed using a two‐sided Student's *t*‐test with Tukey's multiple comparison test.

To verify the enhanced ICD induced by CXB@Hf‐ALN combined with radiotherapy in OS, tumor tissues were analyzed by immunofluorescence co‐staining for the ICD markers calreticulin (CRT) and high mobility group box 1 (HMGB1). Compared with the Blank and RT groups, both the Hf‐ALN+RT and CXB@Hf‐ALN+RT groups exhibited markedly increased CRT exposure on tumor cells, while HMGB1 staining showed a corresponding reduction in nuclear localization, indicating enhanced HMGB1 release (Figure 8a,b). These results demonstrate that CXB@Hf‐ALN combined with radiotherapy induces a stronger ICD response in OS tissues, thereby promoting the release of tumor‐associated antigens and damage‐associated molecular patterns that initiate antitumor immune responses.

To further investigate immune modulation within the tumor microenvironment, COX‐2 expression was evaluated by immunofluorescence staining and PGE_2_ levels were quantified using ELISA. Compared with the Blank group, COX‐2 expression was increased in both the RT and Hf‐ALN+RT groups, consistent with the inflammatory response induced by irradiation. In contrast, COX‐2 expression was markedly reduced in the CXB@Hf‐ALN+RT group due to the release of celecoxib, leading to significant suppression of PGE_2_ production (Figure 8c,d and Figure S18). These findings indicate that CXB effectively inhibits the COX‐2/PGE_2_ immunosuppressive pathway, thereby alleviating radiation‐induced immune suppression and creating a favorable microenvironment for immune activation.

The maturation of dendritic cells (DCs) in orthotopic OS tissues was further quantified by flow cytometry. We have constructed the full sequential gating strategy and added representative plots for every treatment group (Figures S19–S21). Using a sequential gating strategy (live CD45^+^ → CD11c^+^IA/IE^+^ DCs → CD80^+^CD86^+^ mature DCs), the proportion of mature DCs was significantly increased in the Hf‐ALN+RT group (65.4%) compared with the Blank (33.8%) and RT groups (39.4%). Importantly, this effect was further enhanced in the CXB@Hf‐ALN+RT group (75.8%) (Figure 8e,f). These results indicate that Hf‐mediated radiosensitization, together with CXB‐mediated COX‐2 inhibition, promotes DC maturation and enhances antigen presentation. Regulatory T cells (Tregs) were identified by sequential gating of viable CD45^+^CD3^+^CD4^+^FOXP3^+^ cells (Figure 8g,h). The proportion of Tregs was significantly reduced in the Hf‐ALN+RT group (13.1%) compared with the Blank and RT groups, and further decreased in the CXB@Hf‐ALN+RT group (5.25%), indicating effective suppression of immunosuppressive T‐cell populations. By comparing Hf‐ALN+RT with CXB@Hf‐ALN+RT, it can be determined that CXB is a selective COX‐2 inhibitor [41]. As these groups contain the same Hf‐ALN framework and differ mainly in CXB incorporation. CXB loading markedly reduced COX‐2 expression and PGE_2_ production, increased mature CD80^+^ CD86^+^ dendritic cells from 65.4% to 75.8%, and decreased CD4^+^ FOXP3^+^ regulatory T cells from 13.1% to 5.25%. These findings indicate that CXB predominantly alleviates COX‐2/PGE_2_‐mediated immunosuppression and facilitates downstream adaptive immune activation.

Macrophage polarization was also evaluated by flow cytometry (live CD45^+^F4/80^+^ cells), with CD86^+^ and CD206^+^ used to identify M1‐like and M2‐like macrophages, respectively (Figure 8i,j). Treatment with the Hf‐ALN condensate significantly increased the proportion of M1‐like macrophages, indicating that ALN may contribute to reprogramming the immunosuppressive tumor microenvironment. In addition, ELISA analysis showed that secretion of the anti‐inflammatory cytokine IL‐10 was significantly reduced following CXB@Hf‐ALN+RT treatment (Figure S22), further supporting the reprogramming of macrophages toward a pro‐inflammatory phenotype. ALN is not merely a structural ligand for condensate formation but also possesses intrinsic immunomodulatory and anti‐bone resorptive activities. A recent study demonstrated that efficiently delivered ALN can reprogram non‐activated or M2‐like macrophages toward an M1‐like functional state [42, 43]. Our existing results are consistent with these reports. The comparable macrophage‐polarization effects of Hf‐ALN and CXB@Hf‐ALN therefore indicate that ALN is the predominant contributor to M1‐like macrophage repolarization. Moreover, the inhibition of osteoclast activity is a well‐established pharmacological function of ALN [44]. Our data showed that both Hf‐ALN+RT and CXB@Hf‐ALN+RT markedly reduced TRAP‐positive osteoclasts and preserved bone structure, further supporting the anti‐osteoclast activity of ALN. Although the present study does not directly establish the functional necessity of macrophages, the biological interpretation of our findings is supported by a substantial body of current evidence. M1‐like or pro‐inflammatory tumor‐associated macrophages are widely recognized as having antitumor properties through enhanced tumor‐cell phagocytosis, production of inflammatory mediators, antigen presentation, and activation or recruitment of antitumor T‐cell responses [45, 46]. Conversely, M2‐like tumor‐associated macrophages generally promote immune suppression, tumor progression, angiogenesis, and resistance to therapy [47, 48]. Accordingly, therapeutic reprogramming of immunosuppressive tumor‐associated macrophages toward an M1‐like state has become an established strategy for restoring antitumor immunity and converting immunologically “cold” tumors into more inflamed and treatment‐responsive “hot” tumors [49]. Recent mechanistic evidence further demonstrates that M1‐like macrophage repolarization is associated with increased antigen presentation, macrophage phagocytic activity, CD8^+^ T‐cell engagement, and improved tumor control [50, 51].

Activation of DCs and cytotoxic T cells following ICD induction has the potential to generate systemic antitumor immunity. To evaluate whether such systemic immune responses were induced, lung metastasis was examined by H&E staining. Extensive metastatic lesions were observed in the lungs of mice from the Blank and RT groups. Although scattered metastatic foci remained detectable in the Hf‐ALN+RT group, almost no metastatic lesions were observed in the CXB@Hf‐ALN+RT group (Figure 8k and Figure S23). These findings suggest that CXB@Hf‐ALN combined with radiotherapy not only suppresses the primary tumor but also activates systemic immune responses capable of inhibiting distant metastatic progression, consistent with an abscopal antitumor effect. These coordinated immune responses activate systemic antitumor immunity and contribute to the inhibition of osteosarcoma recurrence and pulmonary metastasis, highlighting the potential of this strategy to induce abscopal therapeutic effects.

### 2.9 RNA Sequencing Reveals the Molecular Mechanisms of CXB@Hf‐ALN+RT in Orthotopic Osteosarcoma

**FIGURE 9:**
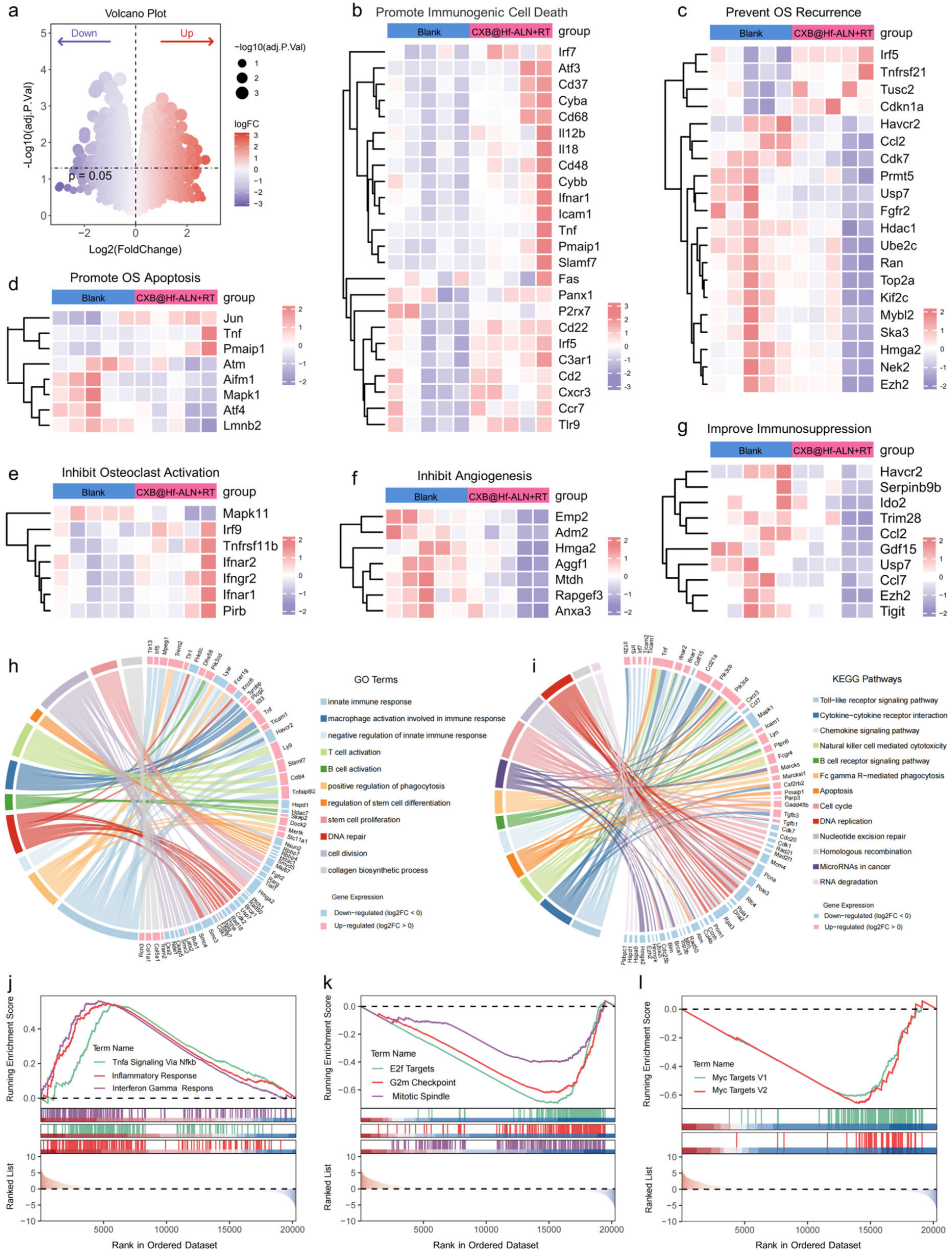
Analysis of Differentially Expressed Genes and Pathway Enrichment in Osteosarcoma. (a) Volcano plot presenting differentially expressed genes (DEGs) identified using the limma package, with a significance threshold of *p* < 0.05, *n* = 5. (b) Heatmap of ICD‐related genes, *n* = 5. (c) Heatmap of recurrence‐associated genes, *n* = 5. (d) Heatmap of apoptosis‐related genes, *n* = 5. (e) Heatmap of osteoclast differentiation‐related genes, *n* = 5. (f) Heatmap of angiogenesis‐related genes, *n* = 5. (g) Heatmap of immunosuppressive genes, *n* = 5. (h) Chord diagram illustrating the results of Gene Ontology (GO) enrichment analysis for the DEGs, *n* = 5. (i) Chord diagram illustrating the results of Kyoto Encyclopedia of Genes and Genomes (KEGG) pathway enrichment analysis for the DEGs, *n* = 5. (j–l) Gene Set Enrichment Analysis (GSEA) results for Hallmark gene sets are displayed, *n* = 5.

To further elucidate the molecular mechanisms underlying the therapeutic effects of CXB@Hf‐ALN combined with radiotherapy, RNA sequencing (RNA‐seq) was performed on tumor tissues from orthotopic OS models. Compared with the untreated Blank group, tumors from the CXB@Hf‐ALN+RT group exhibited substantial transcriptomic alterations across multiple signaling pathways associated with tumor progression and immune regulation (Figure 9). Differential gene expression analysis identified a number of significantly regulated genes (p < 0.05) in the treatment group (Figure 9a), indicating that CXB@Hf‐ALN+RT markedly reshaped the tumor transcriptional landscape. Functional analysis revealed several key biological effects. First, genes associated with ICD were significantly upregulated (Figure 9b), suggesting that the treatment enhances tumor immunogenicity and facilitates the activation of antitumor immune responses. Second, genes associated with tumor recurrence were downregulated (Figure 9c), while apoptosis‐related genes were upregulated (Figure 9d), indicating that the therapy promotes tumor cell apoptosis while potentially reducing the risk of recurrence.

In addition, genes related to osteoclast differentiation were significantly downregulated (Figure 9e), suggesting that CXB@Hf‐ALN+RT may suppress abnormal osteoclast activation within the tumor microenvironment, thereby helping preserve bone integrity and prevent osteolytic destruction. Moreover, the expression of angiogenesis‐related genes (Figure 9f) and immunosuppressive genes (Figure 9g) was also reduced, indicating that the treatment may inhibit tumor vascularization while simultaneously alleviating immunosuppressive signaling within the tumor microenvironment. Pathway enrichment analyses further supported these observations. Gene Ontology (GO) and KEGG pathway analyses revealed that the differentially expressed genes were significantly enriched in biological processes associated with apoptosis, immune responses, angiogenesis regulation, and osteoclast differentiation (Figure 9h,i). Furthermore, Gene Set Enrichment Analysis (GSEA) based on Hallmark gene sets confirmed significant enrichment of pathways related to ICD activation, angiogenesis inhibition, and suppression of immunosuppressive signaling in the CXB@Hf‐ALN+RT group (Figure 9j–l).

Collectively, these transcriptomic results suggest that CXB@Hf‐ALN combined with radiotherapy exerts a multifaceted antitumor effect by simultaneously modulating multiple tumor‐ and immune‐related pathways. Specifically, the treatment: (i) enhances ICD, thereby promoting immune recognition of tumor cells; (ii) suppresses pro‐recurrence, pro‐angiogenic, and immunosuppressive gene programs; (iii) promotes tumor cell apoptosis; and (iv) inhibits osteoclast differentiation, helping maintain bone homeostasis.

## 3 Conclusions

Surgical resection remains the cornerstone of osteosarcoma treatment; however, residual tumor cells and micrometastatic lesions frequently drive post‐surgical recurrence and distant metastasis, which remain the leading causes of patient mortality [52, 53]. Adjuvant radiotherapy (RT) is commonly employed to eliminate postoperative residual disease via ionizing radiation‐induced DNA damage and reactive oxygen species (ROS) generation [54]. Nevertheless, conventional radiosensitizers face severe limitations, including rapid systemic clearance, poor local retention, a lack of immunoregulatory capacity, and off‐target toxicity [55]. Furthermore, standard strategies focus almost exclusively on local tumor destruction while overlooking the immunosuppressive tumor microenvironment (TME), ultimately restricting systemic antitumor immunity [56]. Developing an advanced radiosensitizer that simultaneously amplifies radiotherapy efficacy and remodels the local immune landscape remains a critical hurdle in osteosarcoma management.

To address this challenge, we developed an injectable, TME‐responsive CXB@Hf‐ALN supramolecular condensate for post‐surgical osteosarcoma therapy. Formed through coordination‐driven self‐assembly between Hf^4+^ ions and alendronate (ALN), and further loaded with celecoxib (CXB), this platform enables prolonged local retention within postoperative bone defects and releases its therapeutic payloads in response to the acidic TME. Upon X‐ray irradiation, the high‐Z hafnium (Hf) component enhances radiation energy deposition to accelerate ROS generation, thereby amplifying DNA damage and triggering robust ICD in remaining osteosarcoma cells.

Crucially, the sustained release of CXB and ALN from the condensate acts as a powerful local immunomodulatory platform to dismantle the TME's immunosuppressive barriers. Released CXB effectively inhibits the COX‐2/PGE_2_ signaling pathway‐a major obstacle in RT that typically hinders dendritic cell maturation and cytotoxic T‐cell activation while promoting regulatory Tregs infiltration [57, 58, 59]. Concurrently, the ALN component drives the repolarization of tumor‐associated macrophages toward the pro‐inflammatory M1 phenotype. Together, this coordinated action significantly enhances dendritic cell maturation, promotes CD8^+^ T‐cell activation, and reduces Tregs infiltration, successfully converting a “cold” postoperative TME into an immunologically “hot” environment.

Importantly, the intense ICD induced by the CXB@Hf‐ALN‐sensitized RT provides a continuous source of tumor‐associated antigens and damage‐associated molecular patterns (DAMPs) to initiate a systemic immune response. This robust immune activation extends beyond the primary surgical site to suppress distant pulmonary metastases, which are characteristically lethal in osteosarcoma. The marked inhibition of lung metastatic lesions demonstrates that this localized, synergistic therapy successfully triggers a systemic, immune‐mediated regression of distant tumors.

In summary, the injectable CXB@Hf‐ALN supramolecular condensate successfully bridges the gap between localized radiosensitization and systemic immunotherapy. By pairs‐matching physics‐driven radiation enhancement with targeted biochemical TME remodeling, this platform offers a highly promising and translationally viable strategy to prevent local recurrence, eradicate distant metastases, and improve long‐term outcomes in post‐surgical osteosarcoma therapy.

## 4 Experimental Section

### 4.1 Patient Samples

Clinical OS tissue samples, including primary tumor samples obtained prior to surgery and recurrent tumor samples obtained after surgery, were collected from the Third Affiliated Hospital of Southern Medical University. Prior to undergoing surgery, all participants provided written informed consent. The samples were approved by the Clinical Research Ethics Committee of the Third Affiliated Hospital of Southern Medical University (Ethics review numbers: 2026‐ER‐005). Hematoxylin and eosin (HE) staining and immunofluorescence staining were performed on the tissue samples.

### 4.2 Molecular Dynamics Simulations

In the present work, the structural arrangement of Hf^4^^+^ ions and alendronate molecules in solution was investigated through molecular dynamics (MD) simulations. The composition of each simulated system is provided in Table S2. All simulations were carried out using the Gromacs‐2023.5 software package, with the General AMBER Force Field 2 (GAFF2) applied to describe interatomic interactions. Results were visualized using the Visual Molecular Dynamics (VMD) software.

Non‐bonding interactions, including both van der Waals and electrostatic terms, were treated with a cut‐off radius of rc = 1.2 nm. Interparticle interactions were modeled using the Lennard‐Jones potential.

(1) $$V_{LJ}=\left\{\begin{array}{cc} 4\int \left[{\left(\frac{\sigma }{r}\right)}^{12}-{\left(\frac{\sigma }{r}\right)}^{6}\right], & r<r_{c}\\ 0, & r\geq r \end{array}\right.$$

where *ε* and *σ* denote the strength of the inter‐particle interaction and the characteristic particle size, respectively, both determined by the chosen force field. The electrostatic interaction between particles is described by the Coulomb potential:

(2) $$V_{\mathrm{Coulomb}}=\left\{\begin{array}{cc} \frac{1}{4\pi \varepsilon_{0}}\frac{q_{i}q_{j}}{\varepsilon_{r}r}, & r<r_{c}\\ 0, & r\geq r_{c} \end{array}\right.$$

where *ε_0_* and *ε_r_* denote the vacuum permittivity and the relative dielectric constant, respectively, and *q* represents the charge of the particle. Atomic charges were derived using the Restrained Electrostatic Potential (RESP) method. Long‐range electrostatic interactions were handled with the Particle Mesh Ewald (PME) method.

The simulation protocol began with energy minimization using the steepest descent algorithm, with convergence criteria set to 0.01 kJ/mol for energy and 1000 kJ/mol/nm for forces. Subsequently, molecular dynamics (MD) was carried out in the NVT ensemble for 125 ps (125 000 steps of 1 fs) using the Berendsen thermostat. Newton's equations of motion were integrated with the velocity‐Verlet algorithm.

This was followed by an NPT equilibration phase for 500 ps (250 000 steps of 2 fs), employing the Berendsen algorithm for both temperature and pressure control. Finally, a production MD simulation was conducted for 100 ns (50 000 000 steps of 2 fs) under the NPT ensemble, using the V‐rescale thermostat and the C‐rescale barostat for temperature and pressure coupling, respectively. The time constants for temperature and pressure coupling were set to 1 ps and 2 ps. All simulations were performed under periodic boundary conditions (PBC) at a temperature of 298.15 K and a pressure of 1 atm.

### 4.3 Preparation of CXB@Hf‐ALN

A 25 mL aqueous solution of hafnium tetrachloride (HfCl_4_, 0.04 M, 99.5%; Aladdin, China) and a 25 mL aqueous solution of alendronate sodium trihydrate (ALN, 0.04 M, 97%; Aladdin, China) were prepared separately. The two clear solutions were slowly combined under stirring, resulting in the immediate formation of a milky‐white suspension. The resulting mixture was continuously stirred for 3 h and subsequently centrifuged. After removal of the supernatant, the milky‐white supramolecular condensate was collected and designated as Hf‐ALN.

1 mL of a celecoxib solution in dimethyl sulfoxide (CXB, 0.6 mM, 99%; Aladdin, China) was introduced into the precursor mixture, corresponding to an Hf:ALN:CXB feed mass ratio of 28.1:28.5:1. The mixture was then subjected to the same stirring and centrifugation procedures described above, and the resulting condensate was collected and designated as CXB@Hf‐ALN.

### 4.4 Characterization of CXB@Hf‐ALN

The size variation of the mixed solution was monitored by dynamic light scattering (DLS; Malvern Instruments, Worcestershire, UK). The morphological characteristics and elemental distribution of CXB@Hf‐ALN were systematically investigated using high‐resolution transmission electron microscopy (HR‐TEM; Talos F200S G2S/TEM, Thermo Fisher Scientific) coupled with energy‐dispersive X‐ray spectroscopy (EDS; Talos F200S G2S/TEM, Thermo Fisher Scientific). The crystalline structure of CXB@Hf‐ALN was characterized by X‐ray diffraction analysis (XRD; SmartLab 9KW, Rigaku). The chemical functional groups present in CXB@Hf‐ALN were characterized using Fourier‐transform infrared spectroscopy (FTIR; TENSOR27, Bruker). The secondary structure and conformational characteristics of CXB@Hf‐ALN were analyzed using circular dichroism spectroscopy (CD; Applied Photophysics, Chirascan). The elemental composition, chemical states, and valence band spectrum of CXB@Hf‐ALN were investigated using X‐ray photoelectron spectroscopy (XPS; K‐Alpha, Thermo Fisher Scientific). Absorption spectra of the material were acquired using a solid‐state dedicated ultraviolet‐visible‐near‐infrared spectrophotometer (UV–vis–NIR; Model UV3700i, Shimadzu). The work function of the material was determined by ultraviolet photoelectron spectroscopy (UPS) performed on an Escalab 250Xi system (Thermo Fisher Scientific). The types of ROS (^1^O_2_ and •OH) produced were analyzed using electron spin resonance (ESR; EMXplus‐10/12, Bruker) signals.

### 4.5 Rheological Analysis

The rheological properties of the condensates were systematically characterized using a rotational rheometer (MCR 301, Anton Paar, Austria) equipped with a parallel‐plate geometry (25 mm diameter) at 37 ± 0.1°C. Frequency sweep measurements were performed within the linear viscoelastic region (strain amplitude = 1.0%) over an angular frequency range of 0.1‐10 rad/s to evaluate the mechanical properties and determine the complex viscosity (η*). Strain amplitude sweep tests (γ = 0.1%–10000%) were conducted at a fixed frequency of 1 Hz to identify the critical strain point, which delineates the transition from linear to nonlinear viscoelastic behavior. To assess the self‐healing capability, thixotropic recovery tests were performed by alternating low (γ = 1.0%, 100 s) and high (γ = 100%, 100 s) strain amplitudes at a constant frequency of 1 Hz, with the storage (G′) and loss (G″) modulus being monitored throughout the cyclic deformation process.

### 4.6 Determination of Encapsulation Efficiency and Drug‐loading Content of CXB in Condensate

The content of CXB in the supernatant of the CXB@Hf‐ALN sample after centrifugation was determined by HPLC. Encapsulation efficiency (EE) and drug‐loading content (DLC) were calculated as follows:

(3) $$\begin{array}{c} \mathrm{EE}\;(\%)=\frac{\mathrm{Mass}\;\mathrm{of}\;\mathrm{CXB}\;\mathrm{recovered}\;\mathrm{in}\;\mathrm{CXB}@\mathrm{Hf}-\mathrm{ALN}}{\mathrm{Mass}\;\mathrm{of}\;\mathrm{CXB}\;\mathrm{initially}\;\mathrm{fed}}\;\times \;100\% \end{array}$$

(4) $$\begin{array}{c} \mathrm{DLC}\;(\%)=\frac{\mathrm{Mass}\;\mathrm{of}\;\mathrm{CXB}\;\mathrm{recovered}\;\mathrm{in}\;\mathrm{CXB}@\mathrm{Hf}-\mathrm{ALN}}{\mathrm{Dry}\;\mathrm{mass}\;\mathrm{of}\;\mathrm{CXB}@\mathrm{Hf}-\mathrm{ALN}}\;\times \;100\% \end{array}$$

### 4.7 Extracellular ROS, •OH, and ^1^O_2_ Generation

The total ROS production under varying radiation doses (1, 4, 8, and 10 Gy) was quantified using a ROS detection kit (Beyotime Biotechnology, S0033S). Based on the results, an 8 Gy radiation dose was selected to evaluate ROS generation under radiotherapy conditions in the presence of Hf‐ALN or CXB@Hf‐ALN. The production of singlet oxygen (^1^O_2_) by CXB@Hf‐ALN was assessed using a singlet oxygen sensor green (SOSG) detection kit at the 8 Gy radiation dose. Additionally, hydroxyl radical (•OH) generation was confirmed using disodium terephthalate (TA) as a specific fluorescent probe. TA reacts with •OH to form 2‐hydroxy terephthalate (TAOH), which exhibits distinct fluorescence properties. Briefly, a certain volume of CXB@Hf‐ALN were added to 5 mM TA solution, and the fluorescence intensity of TAOH was measured under radiotherapy conditions (8 Gy) using a fluorescence spectrophotometer (λ_ex_ = 315 nm; λ_em_ = 435 nm).

### 4.8 pH‐Triggered Release of Hf^4+^, ALN and CXB from CXB@Hf‐ALN

The in vitro release kinetics were systematically investigated using a Transwell diffusion system under simulated physiological environments, with PBS buffers at varying pH levels (4.5, 6.8, and 7.4) maintained at 37°C to mimic different biological conditions. Briefly, 200 µL of CXB@Hf‐ALN suspension was introduced into the upper chamber, while the release medium in the lower chamber was collected at predetermined time intervals (6 h, 12 h, 1 day, 3 days, 5 days, 7 days, 14 days and 21days). Quantitative analysis was performed using two analytical techniques: the concentration of Hf^4+^ was determined by inductively coupled plasma mass spectrometry (ICP‐MS; ICAP RQ, Thermo Fisher Scientific, USA), whereas the released amounts of ALN and CXB were quantified by high‐performance liquid chromatography (HPLC; ACQUITY UPLC H‐CLASS, Waters, USA).

### 4.9 Cells Preparation

K7 murine OS cell line (K7M2‐wt, CRL‐2836) was purchased from Shanghai Cell Bank, Chinese Academy of Sciences. K7M2‐wt cells were cultured in high‐glucose DMEM containing 10% FBS and 1% penicillin‐streptomycin.

### 4.10 TAMs, DCs and T Cells Isolation With Magnetic Beads

This method involves obtaining a single‐cell suspension from osteosarcoma tissues of different treatment groups via enzymatic (Tumor Dissociation Kit, mouse, 130‐096‐730, Miltenyi) digestion. TAMs, DCs, and T cells are specifically labeled using im munomagnetic beads conjugated with anti‐F4/80 antibodies (anti‐F4/80 MicroBeads, 130‐110‐443, Miltenyi), anti‐CD11c antibodies (CD11c MicroBeads, 130‐125‐835, Miltenyi), and Pan T Cell Isolation Kit II, (mouse, 130‐095‐130, Miltenyi) respectively. The labeled cells are incubated in a magnetic field, allowing target cells to be retained by magnetic adsorption while unlabeled cells are washed away. Finally, the magnetic field is removed, and the target cells are eluted, yielding a highly purified population for subsequent investigations.

### 4.11 Cytotoxicity Analysis

K7M2‐wt cells were seeded in 96‐well culture plates at an initial density of 0.25 × 10^4^ cells per well. After waiting for the cells to adhere to the wall, the material was added and continued to culture for 6 h, and then received radiotherapy. The cultures were randomly allocated into four groups: (i) untreated control (Blank); (ii) radiotherapy alone (RT); (iii) Hf‐ALN combined with radiotherapy (Hf‐ALN+RT); (iv) CXB@Hf‐ALN combined with radiotherapy (CXB@Hf‐ALN+RT). All groups except the Blank control were subjected to X‐ray irradiation (8 Gy, 30 min) using a clinical linear accelerator. The absorbance of K7M2‐wt cells at Day 1, Day 3 and Day 5 time nodes was detected by CCK8 kit combined with enzyme marker to reflect the cytotoxicity of the material combined with radiotherapy. The cytotoxicity of the materials in combination with radiotherapy were assessed by measuring the absorbance of K7M2‐wt cells at Day 1, Day 3, and Day 5 intervals using a CCK‐8 assay kit in conjunction with a microplate reader.

Macrophages were seeded in 96‐well culture plates at an initial density of 0.25 × 10^4^ cells per well. After waiting for the cells to adhere to the wall, the material was added and continued to culture for 6 h. The cultures were randomly allocated into three groups: (i) Blank; (ii) Hf‐ALN; (iii) CXB@Hf‐ALN. The cytotoxicity of the materials were assessed by measuring the absorbance of Macrophages at Day 1, Day 3, and Day 5 intervals using a CCK‐8 assay kit in conjunction with a microplate reader (Bio‐Rad).

### 4.12 Dead/Live Staining

K7M2‐wt cells were seeded in 24‐well culture plates at an initial density of 1 × 10^4^ cells per well. The samples were divided into four groups after cell adhesion: (i) Blank; (ii) RT; (iii) Hf‐ALN+RT; (iv) CXB@Hf‐ALN+RT. After culture for a while, cell viability and cytotoxicity were evaluated using the Calcein/PI cell viability and cytotoxicity assay kit (Beyotime Biotechnology, C2015S). Fluorescence imaging was performed using an inverted fluorescence microscope (Observer Z1, Carl Zeiss). Quantitative analysis of fluorescence intensity was conducted using ImageJ software.

### 4.13 Colony Formation Assay

K7M2‐wt cells were seeded into cell culture dishes (⌀ = 90 mm) at a density of 1,000 cells per well. Following cell adhesion, the samples were divided into four treatment groups: (i) Blank; (ii) RT; (iii) Hf‐ALN+RT; (iv) CXB@Hf‐ALN+RT. The culture was maintained until distinct colonies became clearly visible in the control group. Subsequently, the colonies were fixed with 4% paraformaldehyde for 30 min and stained with crystal violet for 10 min. Representative images of the colonies were captured, and the number of colonies was quantitatively analyzed.

### 4.14 Immunofluorescence (IF) Staining

Cells were fixed with 4% formaldehyde at 4°C for 30 min, permeabilized with 0.1% Triton X‐100 in PBS, and blocked with 1% BSA in PBS for 2 h. Primary antibody incubation were performed overnight at 4°C (Table S3), followed by secondary antibody treatment at room temperature. Nuclei were stained with DAPI. Next, the images were captured using high‐resolution confocal laser scanning microscope (CLSM; LSM800 with airyscan, Zeiss). Quantitative analysis was conducted using ImageJ software.

### 4.15 Evaluation the CRT and HMGB1 Expression

K7M2‐wt cells were seeded in confocal petri dishes (the glass bottom diameter is 15 mm) at an initial density of 1 × 10^4^ cells per well. The samples were divided into four groups after cell adhesion: (i) Blank; (ii) RT; (iii) Hf‐ALN+RT; (iv) CXB@Hf‐ALN+RT. Then, Anti‐CRT and Anti‐HMGB1 as primary antibody were used for IF staining to evaluation the CRT and HMGB1 expression.

### 4.16 Measurement of ATP Release

K7M2‐wt cells were seeded in 96‐well culture plates at an initial density of 0.25 × 10^4^ cells per well. The samples were divided into four groups after cell adhesion: (i) Blank; (ii) RT; (iii) Hf‐ALN+RT; (iv) CXB@Hf‐ALN+RT. Then, the ATP detection kit (Beyotime Biotechnology, S0026) is used for the test.

### 4.17 Evaluation of Cellular γ‐H2AX

K7M2‐wt cells were seeded in confocal petri dishes at an initial density of 1 × 10^4^ cells per well. The samples were divided into four groups after cell adhesion: (i) Blank; (ii) RT; (iii) Hf‐ALN+RT; (iv) CXB@Hf‐ALN+RT. Then, Anti‐γ‐H2AX as primary antibody were used for IF staining to evaluation of cellular γ‐H2AX.

### 4.18 Intracellular ROS Generation

K7M2‐wt cells were seeded in 24‐well culture plates at an initial density of 1 × 10^4^ cells per well. The samples were divided into four groups after cell adhesion: (i) Blank; (ii) RT; (iii) Hf‐ALN+RT; (iv) CXB@Hf‐ALN+RT. For intracellular ROS detection, cells were incubated with 10 µM 2',7'‐dichlorodihydrofluorescein diacetate (DCFH‐DA; Beyotime Biotechnology, S0033S) in serum‐free medium at 37°C for 60 min. Following three washes with phosphate‐buffered saline (PBS, pH 7.4), fluorescence imaging (λ_ex_ = 488 nm; λ_em_ = 525 nm) was performed using an inverted fluorescence microscope (Observer Z1, Carl Zeiss). Quantitative analysis of fluorescence intensity was conducted using ImageJ software.

### 4.19 ROS Scavenging Assay

To directly determine whether the ICD effects were dependent on ROS, K7M2‐wt cells were treated with CXB@Hf‐ALN+RT in the presence or absence of N‐acetyl‐L‐cysteine (NAC). The DCFH‐DA kit was used to verify that ROS has been scavenging, and then the expressions of CRT and HMGB1 and ATP release were detected.

### 4.20 GSH/GSSG Ratio

K7M2‐wt cells were seeded in 96‐well culture plates at an initial density of 0.25 × 10^4^ cells per well. The samples were divided into four groups after cell adhesion: (i) Blank; (ii) RT; (iii) Hf‐ALN+RT; (iv) CXB@Hf‐ALN+RT. The levels of GSH and GSSG in the cell supernatants after different treatments were measured using GSH and GSSG detection kits (Beyotime Biotechnology, S0053), and the GSH/GSSG ratio was subsequently calculated.

### 4.21 COX‐2 Staining

Macrophages were seeded in confocal petri dishes at an initial density of 1 × 10^4^ cells per well. The samples were divided into four groups after cell adhesion: (i) Blank; (ii) RT; (iii) Hf‐ALN+RT; (iv) CXB@Hf‐ALN+RT. Then, anti‐COX‐2 as primary antibody were used for IF staining to evaluation of cellular COX‐2.

### 4.22 Content of PGE_2_ Assay

Macrophages were seeded in 96‐well culture plates at an initial density of 0.25 × 10^4^ cells per well. The samples were divided into four groups after cell adhesion: (i) Blank; (ii) RT; (iii) Hf‐ALN+RT; (iv) CXB@Hf‐ALN+RT. Then, the PGE_2_ levels in the cell supernatants after different sample treatments were measured using the Prostaglandin E2 ELISA kit (Solarbio, SEKSM‐0034S).

### 4.23 Characterization of Macrophage Polarization

Macrophages were seeded in confocal petri dishes at an initial density of 1 × 10^4^ cells per well, and stimulated with a specified concentration of theophylline to induce M2 polarization. The samples were divided into four groups after cell adhesion: (i) Blank; (ii) RT; (iii) Hf‐ALN+RT; (iv) CXB@Hf‐ALN+RT.

Immunofluorescence staining was performed on macrophages using antibodies Anti‐Arg‐1 and Anti‐iNOS, respectively. After incubation with secondary antibodies, the polarization states of macrophages under different treatment conditions were examined by confocal laser scanning microscopy (CLSM; LSM800 with airyscan, Zeiss).

Macrophages from different groups were stained with antibody panels specific for M1‐like macrophages (F4/80^+^/CD86^+^) and M2 macrophages (F4/80^+^/CD206^+^), and the proportions of each subtype were quantified by flow cytometry (CytoFLEX, Bekcman).

### 4.24 Invasion Assay

Use a Transwell chamber with an 8 µm pore size (24‐well plate). Resuspend 2.5 × 10^4^ cells in serum‐free medium after combined treatment with CXB@Hf‐ALN and radiation, and seed them into the upper chamber pre‐coated with 50 µL of Matrigel. Add DMEM containing 10% FBS to the lower chamber. After 48 h of culture, fix with 4% paraformaldehyde for 30 min, stain with crystal violet, photograph, and count.

### 4.25 T‐Cell‐Mediated Killing of K7M2wt Cells

T cells were isolated from osteosarcoma tissues under different treatment conditions and co‐cultured with K7M2‐wt cells at a ratio of 10:1. After 24 h of co‐culture, the cells were washed with PBS, stained with crystal violet for 10 min, and the staining results were photographed.

### 4.26 CD8^+^ T Cell Exhaustion

As described above, T cells were isolated and resuspended in separation buffer. CD8^+^ T cells were selectively captured using anti‐CD8 antibody‐conjugated magnetic beads (CD8 MicroBeads, cat. no. 130‐116‐478; Miltenyi Biotec). Briefly, the lymphocyte suspension was incubated with anti‐CD8 antibody‐conjugated magnetic beads at 4°C for 15–20 min, followed by magnetic separation. The isolated CD8^+^ T cells were repeatedly stimulated with anti‐CD3 antibody (15 µL/mL) every 48–72 h for a total of six rounds to induce an exhausted CD8^+^ T‐cell phenotype. CD8 staining was performed to evaluate the efficiency of CD8^+^ T‐cell isolation, while PD‐1 and LAG‐3 staining were used to characterize exhaustion‐associated phenotypes. Only samples with a CD8^+^ T‐cell isolation efficiency greater than 90% were included in subsequent analyses.

### 4.27 Osteoclast Differentiation

Macrophages were seeded in confocal petri dishes at an initial density of 1 × 10^4^ cells per well. The samples were divided into four groups after cell adhesion: (i) Blank; (ii) RT; (iii) Hf‐ALN+RT; (iv) CXB@Hf‐ALN+RT. Then, the cell culture medium was replaced with osteoclastogenic induction culture medium (50 ng/mL M‐CSF and 50 ng/mL RANKL). Finally, anti‐TRAP as primary antibody were used for IF staining to evaluate the ability of the condensate to inhibit osteoclast differentiation.

### 4.28 The Biological Safety of CXB@Hf‐ALN

A 10 µL aliquot of indocyanine green (ICG)‐labeled CXB@Hf‐ALN material was injected into the leg of BALB/c mice. The degradation of the condensate was monitored at various time points using a small animal in vivo imaging system (IVIS, ABL X8, Tanon). At the 2‐week mark, the in vivo toxicity of CXB@Hf‐ALN was assessed by measuring liver and kidney biomarkers as well as hematological parameters. After 4 weeks, the mice were euthanized, and major organs including the heart, liver, spleen, lungs, and kidneys were collected for further evaluation of the material's systemic toxicity.

### 4.29 Establishment of Mouse OS In Situ

All mice experiments were conducted in Seyotin Biotechnology Co., Ltd. The experimental protocol was approved by the Animal Ethics Committee, with ethics review numbers SYT2024149. Briefly, BALB/c mice were anesthetized with 2% isoflurane and then 5 µL FBS containing 1 × 10^6^ K7M2‐wt cells were injected into the interosseous membrane adjacent to the tibia using a Hamilton microinjector. After about three weeks, a tumor of the right size forms and is surgically removed. Then all experimental mice were randomly divided into different treatment groups: [i] Blank: no further treatment after tumor resection; [ii] RT: radiotherapy was initiated one day after surgical resection of the tumor, followed by a second session administered one week later; [iii] Hf‐ALN+RT: one day after surgical resection of the tumor, Hf‐ALN condensate was administered followed by radiotherapy. An additional session of radiotherapy was performed one week later; [iv] one day after surgical resection of the tumor, CXB@Hf‐ALN condensate was administered followed by radiotherapy. An additional session of radiotherapy was performed one week later.

### 4.30 Evaluation of the Radiotherapy Effect In Vivo

Following the treatment protocol, mice were randomly selected (*n* = 5 per group) for subsequent analyses. The proximal tibiae were surgically excised and subjected to comprehensive evaluation, including macroscopic imaging, tumor volume measurement (calculated using the formula: V = (length × width^2^)/2), and wet weight determination of both tumor and tumor‐bearing bone specimens. These samples were further processed for MicroCT scanning and histological Analysis to assess the therapeutic efficacy on orthotopic OS.

To investigate the tumor immune microenvironment, an additional cohort of mice (*n* = 5) was sacrificed for tumor dissociation and subsequent flow cytometric analysis of immune cell populations. Furthermore, a separate group of tumor‐bearing mice (n = 5) was maintained for survival analysis to evaluate the long‐term therapeutic outcomes of the treatment regimen.

After the mice in the non‐treatment groups succumbed, lung tissues were harvested from all groups to observe metastatic nodules. The tissues were then subjected to hematoxylin and eosin (H&E) staining for histological analysis.

### 4.31 MicroCT and Analysis

Following 4‐week therapeutic intervention, the tibial specimens were subjected to MicroCT analysis (Bruker, Kontich, Belgium) to evaluate osteolytic alterations. Multiplanar visualization was achieved through DataViewer software, enabling comprehensive assessment in coronal, sagittal, and axial planes. Three‐dimensional morphological reconstruction was performed using CTvox software, while quantitative morphometric analysis was conducted with CTAn software. The quantitative assessment encompassed several key parameters: bone volume ratio (BV/TV, %), bone mineral density (BMD, g/cm^3^), and trabecular structural thickness (Tb.Th, mm).

### 4.32 Histological Analysis

Tibial samples from mice were fixed overnight at 4°C in 10% formalin solution, followed by a one‐week decalcification process and subsequent tissue preparation. All samples were dehydrated using an automated tissue processor (Donatello, DIAPATH) and embedded in paraffin. Serial sections of 4 µm thickness were obtained using a rotary microtome (RM2235, Leica). The sections were subjected to the following staining procedures: immunofluorescence staining with Ki67 antibodiel, TUNEL assay kit (E‐CK‐A325, Elabscience), and γ‐H2AX antibodie to evaluate tumor growth; Goldner's trichrome staining to assess bone tissue regeneration; TRAP staining to analyze osteoclast activation; immunofluorescence staining with CRT/HMGB1 antibodies to evaluate the ICD effect induced by CXB@Hf‐ALN under radiotherapy; and immunofluorescence staining with COX‐2 antibody to assess the ability of CXB@Hf‐ALN to mitigate radiotherapy resistance and immune escape.

### 4.33 In Vivo DC Cells Maturation

Tumor tissues from different treatment groups were harvested and processed into single‐cell suspensions. The cells were sequentially stained with a viability dye, antibodies against CD45 (pan‐immune cells), CD11c and IA/IE (DC cells), as well as CD86 and CD80 (mature DC cells). The proportion of mature dendritic cells was then analyzed by flow cytometry (CytoFLEX, Bekcman). Detailed antibody information is provided in Table S4.

### 4.34 In Vivo Analysis of Treg Cells

Tumor tissues from each experimental group were collected and dissociated into single‐cell suspensions. The cells were sequentially stained with a viability dye, followed by antibody panels against CD45 (pan‐immune cells), CD3 and CD4 (T cells), and finally FOXP3 (regulatory T cells, Tregs). The frequency of Tregs within the CD4^+^ T cell population was quantified using flow cytometry (CytoFLEX, Bekcman). Detailed antibody information is provided in Table S4.

### 4.35 In Vivo Repolarization of M2‐Like to M1‐Like Macrophage‐Phenotype

Tumor tissues from each experimental group were harvested and processed into single‐cell suspensions. The cells were sequentially stained with a viability dye, followed by antibodies against CD45 (pan‐immune cells), F4/80 (macrophages), and then CD86 (M1‐like macrophages) and CD206 (M2‐like macrophages). The proportions of M1‐like (F4/80^+^CD86^+^) and M2‐like (F4/80^+^CD206^+^) macrophage subsets were analyzed by flow cytometry (CytoFLEX, Bekcman). Detailed antibody information is provided in Table S4.

### 4.36 Gating Strategy and Methodology of Flow Cytometry

The specific gating strategies are as follows: (i) debris exclusion by FSC/SSC; (ii) singlet selection using FSC‐A/FSC‐H (and, where available, SSC‐A/SSC‐H); (iii) live‐cell selection; (iv) CD45^+^ leukocytes; and then lineage‐specific gates. Mature DCs are defined within live CD45^+^ CD11c^+^ IA/IE^+^ cells as CD80^+^ CD86^+^; Tregs are defined within live CD45^+^ CD3^+^ CD4^+^ cells as FOXP3^+^; and macrophage subsets are evaluated within live CD45^+^ F4/80^+^ cells using CD86 and CD206.

### 4.37 ELISA of Serum Samples

Residual tumor tissues from the four groups were homogenized and centrifuged to collect supernatants. The concentrations of PGE_2_ and IL‐10 in the supernatants were then determined using mouse PGE_2_ ELISA kits (E‐EL‐0034, Elabscience) and IL‐10 ELISA kits (E‐EL‐M0046, Elabscience).

### 4.38 Bioluminescent (BLI) Imaging

In situ OS modeling was performed using iRFP720 labeled K7M2‐wt cells instead of normal K7M2‐wt cells for bioluminescence imaging. Non‐invasive longitudinal monitoring of tumor progression was performed by scanning mice using bioluminescence mode of a small animal in vivo imaging system (IVIS, ABL X8, Tanon).

### 4.39 RNA Sequencing

RNA samples were extracted one week later from bone tumors subjected to CXB@Hf‐ALN combined radiotherapy and subsequently sent to Personalbio Co., Ltd. for comprehensive transcriptome sequencing and analysis.

### 4.40 Statistical Analysis

This study used OriginPro 2021 and Graphpad Prism v9.0.0 (121) for statistical analysis. Data are presented as mean ± standard deviation (SD). Two‐sided Student's *t*‐test with Tukey's multiple comparison test were used to compare the results among different groups. Results were considered statistically significant if P<0.05. Each experiment was repeated at least five times, yielding highly reproducible results.

## Author Contributions

T.H.X.: conceptualization, data curation, formal analysis, methodology, software, validation, investigation, writing – original draft. Z.L.G.: conceptualization, data curation, methodology, software, formal analysis. C.K.C.: formal analysis, methodology, software. H.Y.: data curation, formal analysis, methodology. Y.C.: data curation, methodology. C.L.: validation, funding acquisition. H.K.H.: software, methodology. Y.L.: data curation. J.Y.G.: software. L.W.: project administration, funding acquisition. C.Y.N.: funding acquisition, resources. G.X.T.: funding acquisition, project administration, supervision, resources, visualization. L.Z.: funding acquisition, investigation, project administration, resources, supervision, visualization, conceptualization, writing – review & editing.

## Conflicts of Interest

The authors declare no conflicts of interest.

## Supporting information




**Supporting File 1**: advs77569‐sup‐0001‐SuppMat.docx.

**Supporting File 2**: advs77569‐sup‐0002‐MovieS1.mp4.

## Data Availability

The data that support the findings of this study are available from the corresponding author upon reasonable request. Raw RNA sequencing data generated in this study have been deposited in the GEO database and are publicly accessible (https://www.ncbi.nlm.nih.gov/geo/query/acc.cgi?acc=GSE325820).

## References

[1] S. Yu and X. Yao , “Advances on Immunotherapy for Osteosarcoma,” Molecular Cancer 23, no. 1 (2024): 192, https://molecular‐cancer.biomedcentral.com/articles/10.1186/s12943‐024‐02105‐9. 39245737 10.1186/s12943-024-02105-9 PMC11382402

[2] M. W. Bishop , “Osteosarcoma,” Hematology/Oncology Clinics of North America 39, no. 4 (2025): 749–760, https://linkinghub.elsevier.com/retrieve/pii/S0889858825000437. 40368742 10.1016/j.hoc.2025.04.005

[3] F. E. Freeman , P. Dosta , L. C. Shanley , et al., “Localized Nanoparticle‐Mediated Delivery of miR‐29b Normalizes the Dysregulation of Bone Homeostasis Caused by Osteosarcoma whilst Simultaneously Inhibiting Tumor Growth,” Advanced Materials 35, no. 23 (2023): 2207877, https://advanced.onlinelibrary.wiley.com/doi/10.1002/adma.202207877. 10.1002/adma.202207877 36994935

[4] J. B. Reinecke , L. J. Garcia , and A. J. Saraf , “Metastasis‐Initiating Osteosarcoma Subpopulations Establish Paracrine Interactions With Lung and Tumor Cells to Create a Metastatic Niche,” Cancer Research 85 (2025): 4341. 40882022 10.1158/0008-5472.CAN-24-3360 PMC12616249

[5] M. Locquet , M. Brahmi , J. Blay , and A. Dutour , “Radiotherapy in Bone Sarcoma: The Quest for Better Treatment Option,” BMC Cancer 23, no. 1 (2023): 742, https://bmccancer.biomedcentral.com/articles/10.1186/s12885‐023‐11232‐3. 37563551 10.1186/s12885-023-11232-3 PMC10416357

[6] X. Wang , K. Zhu , J. Hu , and C. Zhang , “Advances and Challenges in the Treatment of Osteosarcoma,” Progress in Biophysics and Molecular Biology 197 (2025): 60–74, https://linkinghub.elsevier.com/retrieve/pii/S0079610725000367. 40618789 10.1016/j.pbiomolbio.2025.07.001

[7] Y.‐C. Wang , S.‐H. Tsai , M.‐H. Chen , et al., “Mineral Nanomedicine to Enhance the Efficacy of Adjuvant Radiotherapy for Treating Osteosarcoma,” ACS Applied Materials & Interfaces 14, no. 4 (2022): 5586–5597, https://pubs.acs.org/doi/10.1021/acsami.1c21729. 35050587 10.1021/acsami.1c21729

[8] S. Costa and M. R. Reagan , “Therapeutic Irradiation: Consequences for Bone and Bone Marrow Adipose Tissue,” Frontiers in Endocrinology 10 (2019): 587, https://www.frontiersin.org/article/10.3389/fendo.2019.00587/full. 31555210 10.3389/fendo.2019.00587 PMC6727661

[9] A. Piffkó , K. Yang , A. Panda , et al., “Radiation‐Induced Amphiregulin Drives Tumour Metastasis,” Nature 643 (2025): 810, https://www.nature.com/articles/s41586‐025‐08994‐0. 40369065 10.1038/s41586-025-08994-0 PMC13078179

[10] L. Liu , Y. Pan , C. Zhao , et al., “Boosting Checkpoint Immunotherapy With Biomaterials,” ACS Nano 17 (2023): 3225, https://pubs.acs.org/doi/10.1021/acsnano.2c11691. 36746639 10.1021/acsnano.2c11691

[11] Y. Jiang , H. Cao , H. Deng , et al., “Gold‐siRNA Supraclusters Enhance the Anti‐Tumor Immune Response of Stereotactic Ablative Radiotherapy at Primary and Metastatic Tumors,” Nature Biotechnology 43, no. 9 (2025): 1496–1509, https://www.nature.com/articles/s41587‐024‐02448‐0. 10.1038/s41587-024-02448-0 PMC12018592 39448881

[12] W. Zhen , R. R. Weichselbaum , and W. Lin , “Nanoparticle‐Mediated Radiotherapy Remodels the Tumor Microenvironment to Enhance Antitumor Efficacy,” Advanced Materials 35, no. 21 (2023): 2206370, https://advanced.onlinelibrary.wiley.com/doi/10.1002/adma.202206370. 10.1002/adma.202206370 PMC10213153 36524978

[13] A. N. DuRoss , M. R. Landry , C. R. Thomas , M. J. Neufeld , and C. Sun , “Fucoidan‐Coated Nanoparticles Target Radiation‐Induced P‐Selectin to Enhance Chemoradiotherapy in Murine Colorectal Cancer,” Cancer Letters 500 (2021): 208–219, https://linkinghub.elsevier.com/retrieve/pii/S0304383520306182. 33232787 10.1016/j.canlet.2020.11.021 PMC9392493

[14] D. Skrodzki , M. Molinaro , R. Brown , P. Moitra , and D. Pan , “Synthesis and Bioapplication of Emerging Nanomaterials of Hafnium,” ACS Nano 18, no. 2 (2024): 1289–1324, https://pubs.acs.org/doi/10.1021/acsnano.3c08917. 38166377 10.1021/acsnano.3c08917

[15] S. Ding , L. Chen , J. Liao , et al., “Harnessing Hafnium‐Based Nanomaterials for Cancer Diagnosis and Therapy,” Small 19 (2023): 2300341, https://onlinelibrary.wiley.com/doi/10.1002/smll.202300341. 10.1002/smll.202300341 37029564

[16] H. Tian , J. Cao , B. Li , et al., “Managing the Immune Microenvironment of Osteosarcoma: The Outlook for Osteosarcoma Treatment,” Bone Research 11, no. 1 (2023): 11, http://www.ncbi.nlm.nih.gov/entrez/query.fcgi?cmd=Retrieve&db=pubmed&dopt=Abstract&list_uids=36849442&query_hl=1. 36849442 10.1038/s41413-023-00246-z PMC9971189

[17] D. G. DeNardo and B. Ruffell , “Macrophages as Regulators of Tumour Immunity and Immunotherapy,” Nature Reviews Immunology 19, no. 6 (2019): 369–382, https://www.nature.com/articles/s41577‐019‐0127‐6. 10.1038/s41577-019-0127-6 PMC7339861 30718830

[18] W. Liu , L. Li , X. Bai , et al., “Osteosarcoma Cell‐Derived Migrasomes Promote Macrophage M2 Polarization to Aggravate Osteosarcoma Proliferation and Metastasis,” Advanced Science 12 (2025): 2409870, https://advanced.onlinelibrary.wiley.com/doi/10.1002/advs.202409870. 40056029 10.1002/advs.202409870 PMC12061288

[19] F. Cersosimo , S. Lonardi , G. Bernardini , et al., “Tumor‐Associated Macrophages in Osteosarcoma: From Mechanisms to Therapy,” International Journal of Molecular Sciences 21, no. 15 (2020): 5207, https://www.mdpi.com/1422‐0067/21/15/5207. 32717819 10.3390/ijms21155207 PMC7432207

[20] Y. Han , W. Guo , T. Ren , et al., “Tumor‐Associated Macrophages Promote Lung Metastasis and Induce Epithelial‐Mesenchymal Transition in Osteosarcoma by Activating the COX‐2/STAT3 Axis,” Cancer Letters 116 (2019): 440–441, https://linkinghub.elsevier.com/retrieve/pii/S0304383518306256. 10.1016/j.canlet.2018.10.011 30343113

[21] R. Jin , L. Neufeld , and T. L. McGaha , “Linking Macrophage Metabolism to Function in the Tumor Microenvironment,” Nature Cancer 6, no. 2 (2025): 239–252, https://www.nature.com/articles/s43018‐025‐00909‐2. 39962208 10.1038/s43018-025-00909-2

[22] Z. Yu , Y. Peng , J. Gao , et al., “The p23 Co‐Chaperone is a Succinate‐Activated COX‐2 Transcription Factor in Lung Adenocarcinoma Tumorigenesis,” Science Advances 9, no. 26 (2023): ade387, https://go.exlibris.link/VhzxbZgW. 10.1126/sciadv.ade0387 PMC10313168 37390202

[23] N. Markosyan , J. Li , Y. H. Sun , et al., “Tumor Cell–Intrinsic EPHA2 Suppresses Antitumor Immunity by Regulating PTGS2 (COX‐2),” Journal of Clinical Investigation 129, no. 9 (2019): 3594–3609, https://www.jci.org/articles/view/127755. 31162144 10.1172/JCI127755 PMC6715369

[24] W. Guo , J. Luan , X. Huang , et al., “Tumor‐Initiating Stem Cells Fine‐Tune the Plasticity of Neutrophils to Sculpt a Protective Niche,” Cancer Cell 44, no. 1 (2026): 94–111.e11, https://linkinghub.elsevier.com/retrieve/pii/S1535610825004945. 41349542 10.1016/j.ccell.2025.11.001 PMC12700342

[25] M. Morotti , A. J. Grimm , H. C. Hope , et al., “PGE2 Inhibits TIL Expansion by Disrupting IL‐2 Signalling and Mitochondrial Function,” Nature 629, no. 8011 (2024): 426–434, https://www.nature.com/articles/s41586‐024‐07352‐w. 38658764 10.1038/s41586-024-07352-w PMC11078736

[26] S. B. Lacher , J. Dörr , G. P. de Almeida , et al., “PGE2 Limits Effector Expansion of Tumour‐Infiltrating Stem‐Like CD8^+^ T Cells,” Nature 629, no. 8011 (2024): 417–425, https://www.nature.com/articles/s41586‐024‐07254‐x. 38658748 10.1038/s41586-024-07254-x PMC11078747

[27] K. Hayashi , F. Nikolos , Y. C. Lee , et al., “Tipping the Immunostimulatory and Inhibitory DAMP Balance to Harness Immunogenic Cell Death,” Nature Communications 11, no. 1 (2020): 6299, https://www.nature.com/articles/s41467‐020‐19970‐9. 10.1038/s41467-020-19970-9 PMC7721802 33288764

[28] F. Bayerl , P. Meiser , S. Donakonda , et al., “Tumor‐Derived Prostaglandin E2 Programs cDC1 Dysfunction to Impair Intratumoral Orchestration of Anti‐Cancer T Cell Responses,” Immunity 56, no. 6 (2023): 1341–1358.e11, https://linkinghub.elsevier.com/retrieve/pii/S1074761323002285. 37315536 10.1016/j.immuni.2023.05.011

[29] A. E. Badila , D. M. Radulescu , A.‐G. Niculescu , A. M. Grumezescu , M. Radulescu , and A. R. Radulescu , “Recent Advances in the Treatment of Bone Metastases and Primary Bone Tumors: An up‐to‐Date Review,” Cancers 13, no. 16 (2021): 4229, https://go.exlibris.link/rQgNvl8h. 34439383 10.3390/cancers13164229 PMC8392383

[30] J. Zhang , M. Yang , X. Fan , et al., “Biomimetic Radiosensitizers Unlock Radiogenetics for Local Interstitial Radiotherapy to Activate Systematic Immune Responses and Resist Tumor Metastasis,” Journal of Nanobiotechnology 20 (2022): 103, https://jnanobiotechnology.biomedcentral.com/articles/10.1186/s12951‐022‐01324‐w. 35246159 10.1186/s12951-022-01324-w PMC8895626

[31] P. Wen , H. Huang , R. Zhang , et al., “Coacervate Vesicles Assembled by Liquid–Liquid Phase Separation Improve Delivery of Biopharmaceuticals,” Nature Chemistry 17, no. 2 (2025): 279–288, https://www.nature.com/articles/s41557‐024‐01705‐8. 10.1038/s41557-024-01705-8 39806140

[32] T. Liang , Y. Dong , I. Cheng , et al., “In Situ Formation of Biomolecular Condensates as Intracellular Drug Reservoirs for Augmenting Chemotherapy,” Nature Biomedical Engineering 8, no. 11 (2024): 1469–1482, https://www.nature.com/articles/s41551‐024‐01254‐y. 10.1038/s41551-024-01254-y 39271933

[33] P. Zhao , X. Xia , X. Xu , et al., “Nanoparticle‐Assembled Bioadhesive Coacervate Coating With Prolonged Gastrointestinal Retention for Inflammatory Bowel Disease Therapy,” Nature Communications 12, no. 1 (2021): 7162, https://www.nature.com/articles/s41467‐021‐27463‐6. 10.1038/s41467-021-27463-6 PMC8660811 34887414

[34] Y. Lyu , Q. Li , S. Xie , et al., “Synergistic Ultrasound‐Activable Artificial Enzyme and Precision Gene Therapy to Suppress Redox Homeostasis and Malignant Phenotypes for Controllably Combating Hepatocellular Carcinoma,” Journal of the American Chemical Society 147, no. 3 (2025): 2350–2368, https://pubs.acs.org/doi/10.1021/jacs.4c10997. 39723916 10.1021/jacs.4c10997

[35] T. Li , M. Gao , Z. Wu , et al., “Tantalum–Zirconium Co‐Doped Metal–Organic Frameworks Sequentially Sensitize Radio–Radiodynamic–Immunotherapy for Metastatic Osteosarcoma,” Advanced Science 10 (2023): 2206779, https://advanced.onlinelibrary.wiley.com/doi/10.1002/advs.202206779. 36739599 10.1002/advs.202206779 PMC10074130

[36] H. Zhang , M. Cui , D. Tang , et al., “Localization of Cancer Cells for Subsequent Robust Photodynamic Therapy by ROS Responsive Polymeric Nanoparticles with Anti‐Metastasis Complexes NAMI‐A,” Advanced Materials 36 (2024): 2310298, https://advanced.onlinelibrary.wiley.com/doi/10.1002/adma.202310298. 10.1002/adma.202310298 38145801

[37] Z. Huang , S. Huang , S. Song , et al., “Two‐Dimensional Coordination Risedronate‐Manganese Nanobelts as Adjuvant for Cancer Radiotherapy and Immunotherapy,” Nature Communications 15 (2024): 8692, https://www.nature.com/articles/s41467‐024‐53084‐w. 10.1038/s41467-024-53084-w PMC11458765 39375342

[38] Y. Zhang , Y. Liu , S. Luo , et al., “An Adoptive Cell Therapy With TREM2‐overexpressing Macrophages Mitigates the Transition From Acute Kidney Injury to Chronic Kidney Disease,” Clinical and Translational Medicine 15 (2025): 70252, https://onlinelibrary.wiley.com/doi/10.1002/ctm2.70252. 10.1002/ctm2.70252 PMC11859120 40000418

[39] C. Galassi , V. Klapp , T. Yamazaki , and L. Galluzzi , “Molecular Determinants of Immunogenic Cell Death Elicited by Radiation Therapy,” Immunological Reviews 321, no. 1 (2024): 20–32, https://onlinelibrary.wiley.com/doi/10.1111/imr.13271. 37679959 10.1111/imr.13271 PMC11075037

[40] S. Guo , Y. Yao , Y. Tang , et al., “Radiation‐Induced Tumor Immune Microenvironments and Potential Targets for Combination Therapy,” Signal Transduction and Targeted Therapy 8, no. 1 (2023): 205, http://www.ncbi.nlm.nih.gov/entrez/query.fcgi?cmd=Retrieve&db=pubmed&dopt=Abstract&list_uids=37208386&query_hl=1. 37208386 10.1038/s41392-023-01462-z PMC10199044

[41] A. Ruiz‐Saenz , C. E. Atreya , C. Wang , et al., “A Reversible SRC‐relayed COX2 Inflammatory Program Drives Resistance to BRAF and EGFR Inhibition in BRAFV600E Colorectal Tumors,” Nature Cancer 4 (2023): 240–256, https://www.nature.com/articles/s43018‐022‐00508‐5. 36759733 10.1038/s43018-022-00508-5 PMC9970872

[42] L. Kaps , A. Huppertsberg , N. Choteschovsky , et al., “pH‐degradable, Bisphosphonate‐loaded Nanogels Attenuate Liver Fibrosis by Repolarization of M2‐type Macrophages,” Proceedings of the National Academy of Sciences 119, no. 12 (2022): 2122310119, https://pnas.org/doi/full/10.1073/pnas.2122310119. 10.1073/pnas.2122310119 PMC8944276 35290110

[43] M. R. Islam , J. Patel , P. I. Back , et al., “Pegylated Liposomal Alendronate Biodistribution, Immune Modulation, and Tumor Growth Inhibition in a Murine Melanoma Model,” Biomolecules 13, no. 9 (2023): 1309, https://www.mdpi.com/2218‐273X/13/9/1309. 37759709 10.3390/biom13091309 PMC10527549

[44] H. Xu , W. Wang , X. Liu , et al., “Targeting Strategies for Bone Diseases: Signaling Pathways and Clinical Studies,” Signal Transduction and Targeted Therapy 8, no. 1 (2023): 202, http://www.ncbi.nlm.nih.gov/entrez/query.fcgi?cmd=Retrieve&db=pubmed&dopt=Abstract&list_uids=37198232&query_hl=1. 37198232 10.1038/s41392-023-01467-8 PMC10192458

[45] D. J. Kloosterman and L. Akkari , “Macrophages at the Interface of the Co‐Evolving Cancer Ecosystem,” Cell 186, no. 8 (2023): 1627–1651, http://www.ncbi.nlm.nih.gov/entrez/query.fcgi?cmd=Retrieve&db=pubmed&dopt=Abstract&list_uids=36924769&query_hl=1. 36924769 10.1016/j.cell.2023.02.020

[46] S. T. Barry , D. I. Gabrilovich , O. J. Sansom , A. D. Campbell , and J. P. Morton , “Therapeutic Targeting of Tumour Myeloid Cells,” Nature Reviews Cancer 23, no. 4 (2023): 216–237, https://www.nature.com/articles/s41568‐022‐00546‐2. 36747021 10.1038/s41568-022-00546-2

[47] L. Cassetta and J. W. Pollard , “A Timeline of Tumour‐associated Macrophage Biology,” Nature Reviews Cancer 23, no. 4 (2023): 238–257, http://www.ncbi.nlm.nih.gov/entrez/query.fcgi?cmd=Retrieve&db=pubmed&dopt=Abstract&list_uids=36792751&query_hl=1. 36792751 10.1038/s41568-022-00547-1

[48] C. Bleriot , G. Dunsmore , D. Alonso‐Curbelo , and F. Ginhoux , “A Temporal Perspective for Tumor‐Associated Macrophage Identities and Functions,” Cancer Cell 42, no. 5 (2024): 747–758, http://www.ncbi.nlm.nih.gov/entrez/query.fcgi?cmd=Retrieve&db=pubmed&dopt=Abstract&list_uids=38670090&query_hl=1. 38670090 10.1016/j.ccell.2024.04.002

[49] B. Wu , B. Zhang , B. Li , H. Wu , and M. Jiang , “Cold and Hot Tumors: From Molecular Mechanisms to Targeted Therapy,” Signal Transduction and Targeted Therapy 9, no. 1 (2024): 274, http://www.ncbi.nlm.nih.gov/entrez/query.fcgi?cmd=Retrieve&db=pubmed&dopt=Abstract&list_uids=39420203&query_hl=1. 39420203 10.1038/s41392-024-01979-x PMC11491057

[50] S. Ma , B. Sun , S. Duan , et al., “YTHDF2 Orchestrates Tumor‐Associated Macrophage Reprogramming and Controls Antitumor Immunity Through CD8+ T Cells,” Nature Immunology 24, no. 2 (2023): 255–266, https://www.nature.com/articles/s41590‐022‐01398‐6. 36658237 10.1038/s41590-022-01398-6 PMC10150872

[51] F. Zhang , Q. Jiang , J. Cai , et al., “Activation of NOD1 on Tumor‐Associated Macrophages Augments CD8 + T Cell–Mediated Antitumor Immunity in Hepatocellular Carcinoma,” Science Advances 10, no. 40 (2024): adp8266, https://go.exlibris.link/l13c343F. 10.1126/sciadv.adp8266 PMC11446285 39356756

[52] G. S. Brar , A. A. Schmidt , L. R. Willams , M. R. Wakefield , and Y. Fang , “Osteosarcoma: Current Insights and Advances,” Exploration of Targeted Anti‐Tumor Therapy 6 (2025): 1002324, https://www.explorationpub.com/Journals/etat/Article/1002324. 40547807 10.37349/etat.2025.1002324 PMC12179640

[53] H. Jin , C. Huang , Y. Dong , et al., “Research Status and Prospects of Molecular Pathological Mechanisms and Novel Therapeutic Targets of Osteosarcoma: A Systematic Review,” Frontiers in Oncology 15 (2026): 1665299, https://www.frontiersin.org/articles/10.3389/fonc.2025.1665299/full. 41640444 10.3389/fonc.2025.1665299 PMC12864143

[54] H. K. Rachamala , V. S. Madamsetty , R. S. Angom , et al., “Targeting mTOR and Survivin Concurrently Potentiates Radiation Therapy in Renal Cell Carcinoma by Suppressing DNA Damage Repair and Amplifying Mitotic Catastrophe,” Journal of Experimental & Clinical Cancer Research 43, no. 1 (2024): 159, https://jeccr.biomedcentral.com/articles/10.1186/s13046‐024‐03079‐8. 38840237 10.1186/s13046-024-03079-8 PMC11155143

[55] L. Gong , Y. Zhang , C. Liu , M. Zhang , and S. Han , “Application of Radiosensitizers in Cancer Radiotherapy,” International Journal of Nanomedicine 16 (2021): 1083–1102, 10.2147/IJN.S290438. 33603370 PMC7886779

[56] L. Bodei , K. Herrmann , H. Schöder , A. M. Scott , and J. S. Lewis , “Radiotheranostics in Oncology: Current Challenges and Emerging Opportunities,” Nature Reviews Clinical Oncology 19, no. 8 (2022): 534–550, https://go.exlibris.link/rkF2jdCr. 10.1038/s41571-022-00652-y PMC10585450 35725926

[57] O. M. Ozpiskin , L. Zhang , and J. J. Li , “Immune Targets in the Tumor Microenvironment Treated by Radiotherapy,” Theranostics 9, no. 5 (2019): 1215–1231, http://www.thno.org/v09p1215.htm. 30867826 10.7150/thno.32648 PMC6401500

[58] C. Lynch , S. P. Pitroda , and R. R. Weichselbaum , “Radiotherapy, Immunity, and Immune Checkpoint Inhibitors,” The Lancet Oncology 25, no. 8 (2024): e352–e362, https://go.exlibris.link/q8yCY8SX. 39089313 10.1016/S1470-2045(24)00075-5

[59] H. E. Barker , J. T. E. Paget , A. A. Khan , and K. J. Harrington , “The Tumour Microenvironment After Radiotherapy: Mechanisms of Resistance and Recurrence,” Nature Reviews Cancer 15, no. 7 (2015): 409–425, https://www.nature.com/articles/nrc3958. 26105538 10.1038/nrc3958 PMC4896389

