# Supplementary material for: An Injectable Metal‐Drug Supramolecular Condensate Enables Radiosensitization and Systemic Antitumor Immunity for Post‐Surgical Osteosarcoma Therapy
Source: Adv Sci (Weinh). 2026 Aug 31:e77569. Online ahead of print. doi: 10.1002/advs.77569 (PMC13530022; doi:10.1002/advs.77569)
Supplement: Supplementary file 1 [file ADVS-9999-e77569-s002.docx]

**Supporting Information**

**An Injectable Metal-Drug Supramolecular Condensate Enables Radiosensitization and Systemic Antitumor Immunity for Post-surgical Osteosarcoma Therapy**

*Tianhua Xiao**^#^,* *Zunlei Gong^#^,* *Chuke Chen^#^, Hui Yu,* *Yu Cai, Can Liu, Hongkai Huang, Ying Li, Jinyuan Guan, Le Wang^*^, Chengyun Ning, Guoxin Tan^*^, Lei Zhou**^*^*

T. Xiao, H. Huang, Y. Li, J. Guan, Prof. G. Tan

School of Chemical Engineering and Light Industry, Guangdong University of Technology, Guangzhou, 510006, China.

E-mail: [tanguoxin@126.com](mailto:tanguoxin@126.com) (G. Tan)

T. Xiao, Dr. Z. Gong, C. Chen, Dr. H. Yu, Y. Cai, Prof. L. Wang, Prof. L. Zhou

Department of Orthopaedic Surgery, Guangzhou Key Laboratory of Spine Disease Prevention and Treatment, The Third Affiliated Hospital, Guangzhou Medical University, Guangzhou, 510150, China.

E-mail: Email: [wangle8273@163.com](mailto:wangle8273@163.com) (L. Wang); [zhoul@gzhmu.edu.cn](mailto:zhoul@gzhmu.edu.cn) (L. Zhou)

C. Liu

Department of Spine Surgery, Center for Orthopedic Surgery, The Third Affiliated Hospital of Southern Medical University, Guangzhou, 510515, China

Prof. C. Ning

School of Materials Science and Engineering, National Engineering Research Center for Tissue Restoration and Reconstruction, South China University of Technology, Guangzhou, 510641, China.

**Figure S1. Clinical and single-cell bioinformatics analysis.** a) Recurrence-Free survival curve of osteosarcoma patients with high COX-2 expression and low COX-2 expression. b) Progression-Free survival curve of osteosarcoma patients with high COX-2 expression and low COX-2 expression. c) UMAP distribution of cellular clusters across primary and recurrent OS tissues. d) Relative proportions of each cellular cluster in different OS tissue samples (primary, recurrent and metastasis). e) HE staining of primary and recurrent clinical OS tissues, the black arrow represents osteosarcoma tissue, n=5. f) UMAP visualization of different cell types of immune cells in OS tissue. g) Relative proportions of different cell types within the immune cell population in different OS samples (primary, recurrent and metastasis).

**Figure S2. Computational Simulation of ALN and Hf^4+^ Coordination.** a) Electrostatic potential distribution of ALN and Hf^4+^ coordination obtained by density functional theory (DFT) calculations. b) Optimized coordination structure between ALN and Hf^4+^. c) Binding energy of the ALN and Hf^4+^. d) Coordination number of Hf^4+^ in the ALN-Hf^4+^ system.

**Figure S3.** A photograph of the Hf-ALN hydrogel obtained after mixing and stirring aqueous solutions of HfCl_4_ and ALN, followed by centrifugation.

**Figure S4.** Particle size distribution of Hf-ALN at 0, 1.5, and 3 hours.

**Figure S5. X-ray Photoelectron Spectroscopy (XPS) Analysis of Elemental Composition and Chemical States.** a) XPS survey spectrum of the Hf-ALN. b) Elemental composition (atomic %) of the Hf-ALN. c) High-resolution Hf 4f spectrum of the Hf-ALN. d) XPS survey spectrum of the CXB@Hf-ALN. e) Elemental composition (atomic %) of the CXB@Hf-ALN. f) High-resolution Hf4f spectrum of the CXB@Hf-ALN.

**Figure S6.** **Spectroscopic Characterization.** a) Circular dichroism (CD) spectrum of the CXB@Hf-ALN. b) X-ray diffraction (XRD) pattern of the Hf-ALN.

**Figure S7.** The encapsulation efficiency (a) and drug-loading content (b) of CXB in condensate.

**Figure S8.** **Rheological Characterization.** a) Shear-thinning behavior of the Hf-ALN. b) Stability of the Hf-ALN under steady shear. c) Strain amplitude sweep of the Hf-ALN. d) Alternating cyclic strain test (1% and 100% strain) showing the modulus recovery of the Hf-ALN.

**Figure S9. Fluorescence imaging of in vivo Hf-ALN degradation.** a) Representative fluorescence micrographs of BALB/c mice at different time points after intramuscular injection of ICG-labeled Hf-ALN (n=5). b) Fluorescence intensity at different time points.

**Figure S10. The ICD is dependent on the production of ROS.** a) Representative images of ROS staining under different treatment conditions. b) Quantitative analysis of ROS fluorescence intensity. c) Representative IF images of ICD markers (CRT and HMGB1) in K7M2-wt cells under different treatment conditions, n=5. d) Quantification analysis of CRT fluorescence intensity. e) Quantification analysis of HMGB1 fluorescence intensity. f) ATP concentration in the supernatant of K7M2-wt cells under different treatment conditions.

**Figure S11.** **In vitro study of CXB@Hf-ALN inhibiting osteoclastic differentiation of BMDMs.**  a) IF staining of TRAP protein, n=5. b) Quantitative analysis of TRAP fluorescence intensity. c) Quantitative analysis of the number of nuclei per osteoclast.

**Figure S12. The ROS-induced high expression of ICD in K7M2-wt cells and activates the downstream immune response.** a) Schematic illustration of the experimental workflow for investigating the effects of conditioned medium from ROS-induced ICD-high K7M2-wt cells on macrophage phenotypic modulation. b) Representative immunofluorescence images of iNOS and Arg-1 expression in macrophages treated with conditioned medium, n=5. c) Quantitative analysis of iNOS and Arg-1 fluorescence intensity. d) Schematic illustration of the experimental workflow for investigating the effects of conditioned medium from ROS-induced ICD-high K7M2-wt cells on the maturation regulation of DCs cells. e) Representative immunofluorescence images of CD11c expression in DCs cells treated with conditioned medium, n=5. f) Quantitative analysis of CD11c fluorescence intensity. g) Schematic illustration of the experimental workflow for investigating the effects of conditioned medium from ROS-induced ICD-high K7M2-wt cells on the regulation of activated T cells. h) Representative immunofluorescence images of CD8 expression in T cells treated with conditioned medium, n=5. i) Quantitative analysis of CD8 fluorescence intensity.

**Figure S13. CD8^+^ T-cell exhaustion impairs the suppression of K7M2-wt cell activity.** a) IF staining of CD8 in exhausted CD8^+^ T cells, n=5. b) Quantitative analysis of CD8 fluorescence intensity. c) IF staining of PD-1/LAG-3 in exhausted CD8^+^ T cells, n=5. d) Quantitative analysis of PD-1 and LAG-3 fluorescence intensity. e) Schematic diagram of co-culture of CD8^+^ exhausted T cells with K7M2-wt cells. f) Crystal violet staining of co-cultured CD8^+^ exhausted T cells and K7M2-wt cells, n=5. f) Quantification analysis of cell numbers after crystal violet staining.

**Figure S14.** Blood biochemical and routine parameters of mice after treatment.

**Figure S15.** H&E-stained images of the heart, liver, spleen, lungs, and kidneys from each treatment group, n=5.

**Figure S16. MicroCT-based Quantitative Analysis of Bone Quality at the Tumor Site.** a) Analysis of bone mineral density (BMD). b) Analysis of trabecular thickness (Tb.Th).

**Figure S17.** **CXB@Hf-ALN Potentiates Radiotherapy-Induced Tumor Apoptosis.** a) Representative fluorescence images of co-staining for Ki67 (proliferation) and TUNEL (apoptosis) in tumor tissues 21 days post-treatment, n=5. b) Quantitative analysis of Ki67-positive cells. c) Quantitative analysis of TUNEL-positive cells. d) Representative immunofluorescence images of γ-H2AX (DNA damage) expression in tumor sections, n=5. e) Quantitative analysis of γ-H2AX fluorescence intensity.

**Figure S18.** Quantitative analysis of COX-2 immunofluorescence intensity in tibial tumor tissues 21 days after treatment across different groups.

**Figure S19.** Gating strategy plots for the flow cytometric identification of the maturation of dendritic cells, FSC-A/FSC-H → SSC-A/SSC-H → CD45 → CD11c/IAIE → CD80/CD86, n=5.

**Figure S20.** Gating strategy plots for the flow cytometric identification of Treg cells, FSC-H/SSC-H → FSC-A/FSC-H → CD45 → CD3/CD4 → FOXP3, n=5.

**Figure S21.** Gating strategy plots for the flow cytometric identification of M1-like macrophages and M2-like macrophages, FSC-A/FSC-H → SSC-A/SSC-H → CD45 → F4/80 → CD206/CD86, n=5.

**Figure S22.** The level of IL-10 in tumor tissues was measured by ELISA.

**Figure S23.** Quantification of pulmonary metastatic nodules by H&E staining.

**Table S1. The characteristics of the patients**

| Characteristic | Category | Number (n) | Percentage (%) |
| --- | --- | --- | --- |
| Total Cases | - | 73 | - |
| Gender | Male | 41 | 56.16 |
|  | Female | 32 | 43.83 |
| Race | White | 47 | 64.38 |
|  | Black or african american | 6 | 8.22 |
|  | Asian | 6 | 8.22 |
|  | Unknown | 14 | 19.18 |
| Ethnicity | Hispanic or latino | 10 | 13.70 |
|  | Not hispanic or latino | 53 | 72.60 |
|  | Unknown | 10 | 13.70 |
| First_event | Relapse | 38 | 52.05 |
|  | None | 32 | 43.84 |
|  | Dead | 2 | 2.73 |
|  | Second Malignant Neoplasm | 1 | 1.37 |
| Vital_status | Alive | 44 | 60.27 |
|  | Dead | 29 | 39.72 |

**Table S2. The composition of each simulation system**

|  | Alendronate^+^ | Na^+^ | Hf^4+^ | Cl^-^ | Water |
| --- | --- | --- | --- | --- | --- |
| System 1 | 100 | 100 | 100 | 400 | 15219 |

**Table S3.** **Primary antibodies used for immunofluorescence staining**

| **Antibodies** | **Species** | **Dilution ratio** | **Source** |
| --- | --- | --- | --- |
| **CRT** | Rabbit | 1:1000 | Abcam |
| **HMGB1** | Mouse | 1:1000 | Invitrogen |
| **γ-H2AX** | Mouse | 1:1000 | Abcam |
| **COX-2** | Rabbit | 1:1000 | Abcam |
| **Ki67** | Rabbit | 1:1000 | Abcam |
| **iNOS** | Rabbit | 1:500 | Proteintech |
| **Arg-1** | Mouse | 1:500 | Proteintech |
| **CD11c** | Mouse | 1:500 | Thermo |
| **CD8** | Rabbit | 1:500 | Abcam |
| **TRAP** | Rabbit | 1:500 | Abcam |
| **CD68** | Mouse | 1:500 | Abcam |
| **CD206** | Rabbit | 1:500 | Abcam |
| **PD-1** | Mouse | 1:500 | Proteintech |
| **LAG-3** | Rabbit | 1:500 | Proteintech |

**Table S4.** **Antibodies used for flow cytometry**

| **Antibodies** | **Species** | **Dilution ratio** | **Source** |
| --- | --- | --- | --- |
| **F4/80** | Rat | 1:200 | BD Pharmingen |
| **CD86** | Rat | 1:200 | BD Pharmingen |
| **CD206** | Rat | 1:200 | BD Pharmingen |
| **L-D** | / | 1:200 | BD Pharmingen |
| **CD45** | Rat | 1:200 | BD Pharmingen |
| **CD11c** | Hamster | 1:200 | BD Pharmingen |
| **IAIE** | Rat | 1:200 | BD Pharmingen |
| **CD80** | Hamster | 1:200 | BD Pharmingen |
| **CD3** | Hamster | 1:200 | BD Pharmingen |
| **CD4** | Mouse | 1:200 | BD Pharmingen |
| **FOXP3** | Rat | 1:200 | BD Pharmingen |
